# Transcriptome analysis of CpGV in midguts of type II resistant codling moth larvae and identification of contaminant infections by SNP mapping of RNA-Seq data

**DOI:** 10.1128/jvi.00537-24

**Published:** 2024-06-27

**Authors:** Shili Yang, Maximilian Amberger, Jörg T. Wennmann, Johannes A. Jehle

**Affiliations:** 1 Julius Kühn Institute (JKI)—Federal Research Centre for Cultivated Plants, Institute for Biological Control, Dossenheim, Germany; Wageningen University & Research, Wageningen, Netherlands

**Keywords:** *Cydia pomonella*, baculovirus, *Lefavirales*, resistance, *in vivo* infection, gene expression

## Abstract

Various isolates of the Cydia pomonella granulovirus (CpGV) are used as insect pest control agents against codling moth (CM, *Cydia pomonella* L.), a predominant pest in apple orchards. Three different types (I–III) of dominantly inherited field resistance of CM larvae to CpGV have been recently identified. In this study, transcription of virus genes in midgut cells of type II-resistant CM larvae infected with different CpGV isolates, i.e., CpGV-M and CpGV-S (both prone to type II resistance) as well as CpGV-E2 (breaking type II resistance) was determined by strand-specific RNA sequencing (RNA-Seq) at an early infection stage (72 h post infection). Based on principal component analysis of read counts and the quantitative distribution of single nucleotide polymorphisms (SNPs) in the RNA-Seq data, a bioinformatics analysis pipeline was developed for an *a posteriori* identification of the infective agents. We report that (i) identification of infective agent is crucial, especially in *in vivo* infection experiments, when activation of covert virus infections is a possibility, (ii) no substantial difference between CpGV-M and CpGV-S transcription was found in type II-resistant CM larvae despite a different resistance mechanism, (iii) the transcription level of CpGV-M and CpGV-S was much lower than that of CpGV-E2, and (iv) *orf59* (*sod*), *orf89* (*pif-6*)*, orf92* (*p18*), and *orf137* (*lef-10*) were identified as significantly downregulated genes in resistance-prone isolates CpGV-M and CpGV-S. For type II resistance of CM larvae, we conclude that CpGV-M and CpGV-S are both able to enter midgut cells, but viral transcription is significantly impaired at an early stage of infection compared to the resistance-breaking isolate CpGV-E2.

**IMPORTANCE:**

CpGV is a highly virulent pathogen of codling moth, and it has been developed into one of the most successful commercial baculovirus biocontrol agents for pome fruit production worldwide. The emergence of field resistance in codling moth to commercial CpGV products is a threat toward the sustainable use of CpGV. In recent years, different types of resistance (type I–III) were identified. For type II resistance, very little is known regarding the infection process. By studying the virus gene expression patterns of different CpGV isolates in midguts of type II-resistant codling moth larvae, we found that the type II resistance mechanism is most likely based on intracellular factors rather than a receptor component. By applying SNP mapping of the RNA-Seq data, we further emphasize the importance of identifying the infective agents in *in vivo* experiments when activation of a covert infection cannot be excluded.

## INTRODUCTION

The Cydia pomonella granulovirus (CpGV) is a double-stranded DNA virus with a circular genome of 120.8–124.3 kbp in length and containing 137–143 open reading frames (ORFs) (1). It belongs to the species *Betabaculovirus cypomonellae* (family *Baculoviridae*) (2). CpGV is widely used in Europe, Australasia, South Africa, and the Americas to control the codling moth (CM), *Cydia pomonella* L. (Lepidoptera: Tortricidae), a key pest in pome fruit plantations (3–5). CpGV-based biocontrol products are both highly virulent and host specific and are, therefore, environmentally friendly (6).

In 1964, the first CpGV isolate (CpGV-M) was found in Mexico (7). Later on, numerous CpGV isolates were discovered from different regions worldwide (8–13), and seven phylogenetic lineages, namely, the genome groups A to G, were identified (10). CpGV isolates belonging to different lineages vary from each other by indel mutations and single-nucleotide polymorphisms (SNPs). Detailed SNP maps of CpGV are available for more than 20 isolates (14).

For CM control, the isolate CpGV-M (genome group A) had been registered in the late 1980s in Europe and later in many other countries worldwide. After many years of successful use of CpGV in organic and integrated pome fruit production, first reports of field resistance of CM populations to commercial CpGV products appeared in Europe in 2005 (15, 16). Further selection of one of these field populations resulted in a genetically homogenous CM line CpRR1, which showed a monogenic, dominant, and Z-linked inheritance of the resistance, eventually termed type I resistance (17). As concluded by Gebhardt et al. (18), type I resistance of CM was directed to CpGV-M (genome group A) but not to other virus isolates, such as CpGV-S (genome group E) or CpGV-E2 (genome group B), which were able to break type I resistance. The viral gene *pe38* of CpGV was identified as the key factor in overcoming CpGV resistance in codling moth, as CpGV-M contains a 24-nucleotide repeat insertion within *pe38* in CpGV-M, whereas CpGV isolates without this insertion were able to break type I resistance (18). By comparing virus transcription and DNA replication in midguts of type I-resistant CpRR1 larvae, it was further shown that CpGV-M could enter the midgut cells, but the viral gene transcription machinery and viral DNA replication were blocked (19–21).

More recently, a second type of CpGV field resistance (type II), not following the inheritance pattern of type I resistance, was reported (22). Last but not least, further types of CpGV resistance have been observed in the field (23, 24). To further examine type II resistance, a genetically homogeneous inbred strain CpR5M was established (25). In contrast to type I resistance, it showed an autosomal dominant inheritance of resistance, as well as cross resistance to CpGV-M and CpGV-S, but not to CpGV-E2 (25).

For type II-resistant CpR5M larvae, very little is known regarding the infection process and transcription of different CpGV isolates. A recent study demonstrated that resistance of CpR5M is systemic for CpGV-M but midgut-related for CpGV-S, suggesting different resistance mechanisms for the two viral isolates (26).

To shed further light on the infection process of CpGV in the midgut of CpR5M larvae, we have applied a comparative Illumina RNA-Seq for viral transcripts of three different CpGV isolates, i.e., CpGV-M and CpGV-S and the resistance-breaking CpGV-E2, at 72 h post infection (hpi) since this time point was shown in previous experiments to be crucial for the switch from early to late infection (19). When analyzing the RNA-Seq data of inoculations with different CpGV isolates, we noticed the presence of CpGV reads which did not match with the known sequences of the original inoculum, suggesting the activation of covert CpGV infections apparently present in the rearing of CpR5M codling moth. Such covert infections are not unusual for reared insects and can comprise either non-productive latency or sublethal infections involving low-level production of virus progeny (27). Triggered by certain environmental or physiological stressors, covert baculovirus infections can be activated and produce an overt infection and lethal disease (27, 28).

To overcome this challenge of a covert infection to the RNA-Seq analysis, a read-based identification of the infective agent was performed using a method based on the quantitative distribution of single-nucleotide polymorphisms (SNPs) and principal component analysis (PCA) of read counts in the RNA-Seq data set. In addition, an updated method was developed to detect the differentially expressed genes between different virus isolates on the viral transcriptome analysis. The sequencing and analysis methods described here should be broadly applicable for other viruses and hosts.

## RESULTS

### Infection of CpR5M larvae with different CpGV isolates

**Fig 1:**
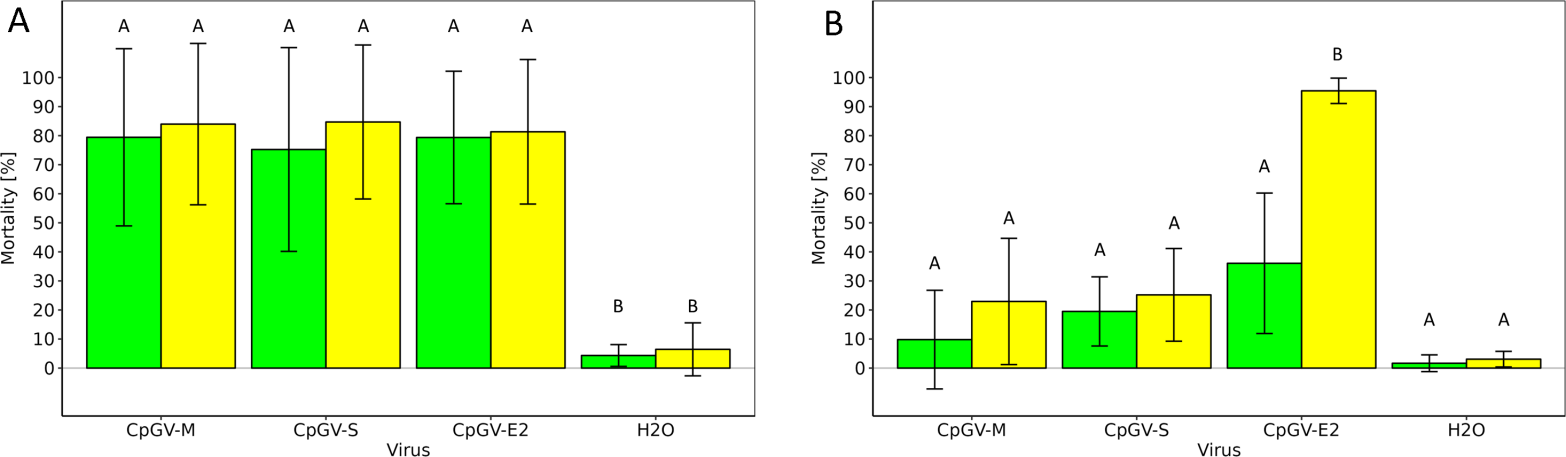
Mean mortality and its standard deviation of three replicates of third instars of CpS (A) and CpR5M (B) infected with 10,000 OBs of either CpGV-M, CpGV-S, or CpGV-E2, the mock infection (H_2_O) was used as negative control. Mortality was recorded at 7 (green columns) and 14 (yellow columns) days post infection (dpi). Different letters indicate statistically different mortality within the two time points of observation [Tukey honestly significant difference (HSD), *P* < 0.05].

Third instars of CpR5M were orally infected with 10^4^ OBs/larva of either CpGV-M, -S, or -E2. Whereas midguts of one cohort of larvae were collected at 72 hpi for transcription analyses, the remaining larvae were further reared and virus-induced mortality was recorded at 7 and 14 days post infection (dpi). As a control, the mortality of the CpGV-susceptible CM strain CpS ranged from 74% to 78% after 7 dpi and 81% to 86% after 14 dpi, without difference between the three CpGV isolates (Fig. 1A). In contrast, mortality of CpR5M larvae depended on the virus inoculum; CpGV-M and CpGV-S caused a lower mortality than the resistant-breaking isolate CpGV-E2 at both 7 and 14 dpi (Fig. 1B). At 14 dpi, mortality caused by CpGV-E2 reached 95%, whereas mortality of larvae treated with CpGV-M and -S was below 25% (Tukey HSD, *P* < 0.05). Mortality of the mock infection control groups (H_2_O) of CpS and CpR5M was always below 5% until 14 dpi (Fig. 1). This experiment proved resistance of CpR5M to CpGV-M and -S but not to CpGV-E2.

### Overview of RNA-Seq data

For comparative transcription analysis of viral genes, RNA samples were prepared from midguts of CpR5M larvae infected with either CpGV-M, -S, or -E2 (and mock infection, H_2_O); three biological replicates (1–3) of each treatment resulted in twelve RNA samples with were individually sequenced using an Illumina NextSeq 500 sequencer.

First, the RNA-Seq reads were mapped against the consensus genome of CpGV-M (GenBank KM217575) as a reference to identify those reads which were of CpGV origin (Table 1). For the samples from larvae infected with CpGV-M, -S, or -E2, the total paired reads (viral and cellular paired reads) ranged from 5.24 million (CpGV-M treatment in replicate M2) to 7.97 million (CpGV-E2 treatment in replicate E3), whereas the sole counts of viral paired reads were between 8,567 (M1) and 319,575 (E3) (Table 1). For infections with CpGV-M and -S, the mean percentage of viral reads was 0.35% and 0.42%, respectively (Table 1).

**TABLE 1:** Total number of paired read and viral paired read counts from midguts of CpR5M larvae infected with CpGV-M, -S, or -E2 and mock infection (H_2_O) at 72 h post infection^*a*^

Virus treatment	CpGV-M			CpGV-S			CpGV-E2			H_2_O		
Replicate	M1	M2	M3	S1	S2	S3	E1	E2	E3	K1	K2	K3
Viral paired reads^*b*^	8,567	9,221	49,425	15,556	29,743	39,172	201,346	150,463	319,575	23	64,637	39
Total paired reads (million)^*c*^	6.40	5.24	5.26	7.32	7.76	5.83	7.91	7.01	7.97	6.29	8.21	5.08
Percent viral paired reads	0.13	0.18	0.94	0.21	0.38	0.67	2.55	2.15	4.01	0.00	0.79	0.00
Mean percent viral paired reads	0.35			0.42			2.90			0.26		
STDEV	0.45			0.23			0.98			0.45		

^*a*^ Given are the numbers for three independent replicates (1–3). STDEV, = standard deviation.

^*b*^ Viral paired read counts identified in three biological replicates of the midgut by mapping filtered reads from each replicate sample to the CpGV genome using HISAT.

^*c*^ Total paired reads (cellular + viral) counts identified in three biological replicates of midgut samples.

Infection with the resistance-breaking isolate CpGV-E2 generated 2.90% viral paired reads, indicating that CpGV-E2 had much higher transcriptional level than CpGV-M and -S. In the mock infection group (H_2_O), only very few viral reads were found for the samples K1 (23 reads) and K3 (39 reads) (Table 1). These reads mapped to eight ORFs for K1 and ORFs for K3, and both samples contained reads of *orf44* (*orf36L*), *orf 57* (*pp31/39K*)*, orf72* and *orf90* (*helicase*) (Table S1). Interestingly, the mock infection sample K2 contained 64,637 reads specific for CpGV, suggesting a contaminant CpGV infection in this sample (Table 1). To understand the presence and origin of the CpGV isolate in K2, this sample was included in the further analyses, whereas K1 and K3 were not further considered due to their neglectable few CpGV reads. Thus, the samples M1-M3, S1-S3, E1-E3, and K2 were further processed.

### Coverage plotting of viral reads

To obtain a global picture of the expression intensity of CpGV genes, coverage plots were generated by mapping the RNA-Seq reads against the genome of CpGV-M (GenBank KM217575) as a reference using BWA-MEM mapper (Fig. S1). The samples M1–M3 and S1–S3 showed very similar coverage patterns and a low viral gene transcription level in all replicates. The read coverage of most genes was above zero and only few genes had no expression without any reads mapped, indicating that many genes of CpGV-M and CpGV-S were transcribed in the midgut of CpR5M, though at very low level. In contrast, the coverage in the samples E1–E3 was highly increased, suggesting that gene expression of the resistance-breaking isolate CpGV-E2 was considerably higher than that of CpGV-M and CpGV-S. For all samples containing one of the CpGV isolates, the positions of read peaks were similar, demonstrating that those genes with highest expression were the same, though at different levels (Fig. S1). The coverage plot of sample K2 indicated viral gene transcription though it was not clear which CpGV genotype was transcribed in this control (Fig. S1).

### Distinguishing CpGV genotypes in RNA-Seq samples based on SNP distribution

**Fig 2:**
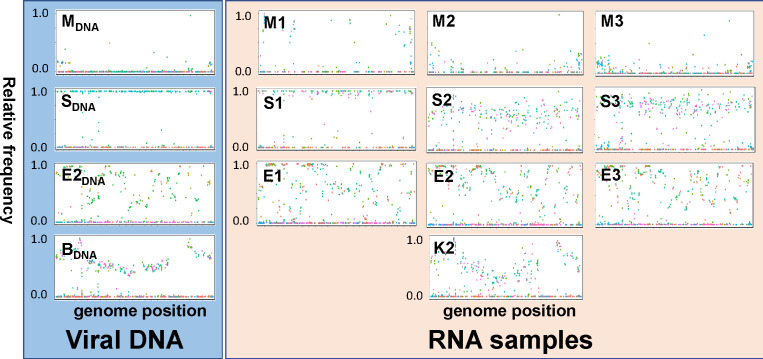
Single nucleotide polymorphism (SNP) frequency plots of viral DNA sequences of CpGV-M (M_DNA_) (top row), CpGV-S (S_DNA_) (second row), CpGV-E2 (E2_DNA_) (third row), and CpGV-B (B_DNA_) (bottom row) and the corresponding biological replicates of RNA-Seq data (samples M1–M3, S1–S3, E1–E3) of each virus and the mock infection sample K2. All data were mapped against CpGV-M genome (KM217575) as a reference. The SNP positions in the genomes (horizontal axis) were plotted against the frequency of the alternative nucleotide (vertical axis). The specificities of SNP positions were marked in blue color for CpGV-M, in magenta for CpGV-S, in red for CpGV-E2, and in green for all three isolates. Note that SNP mapping of sample K2 generates a similar SNP pattern as CpGV-B DNA.

Because of the possibility of covert virus infection or virus contamination, the virus used for inoculating the larvae might not be the same as the virus causing an infection in the experiment. To examine and quantify the viral genotype(s) behind each RNA-Seq sample, the RNA-Seq reads were mapped against the DNA reference sequence of CpGV-M (KM217575) using the bacsnp package (29). As a reference, the SNP frequency plots of the DNAs of CpGV-M, CpGV-S, and CpGV-E2 used for inoculation were included (Fig. 2). Given that the administered virus was identical to the infective agent, similar patterns of isolate-specific SNPs were expected for both DNA-Seq data and RNA-Seq. Indeed, this was the case for the DNA of CpGV-M and the three RNA-Seq samples M1–M3, the DNA of CpGV-E2 and the samples E1–E3, the DNA of CpGV-S and sample S1. In contrast, the RNA-Seq samples S2 and S3 showed a horizontal cloud of SNPs at about 70%. This finding indicated a mixed infection in the samples S2 and S3 with CpGV-S and CpGV-M of about 70% and 30%, respectively (Fig. 2, second row). Strikingly, the SNP pattern of the RNA-Seq data of sample K2 was highly similar to the DNA-Seq pattern of a novel, recently identified CpGV-B variant (Fig. 2, bottom plots), which appears to be an internal virus originating from a covert infection of CpR5M (Yang et al., in preparation).

**Fig 3:**
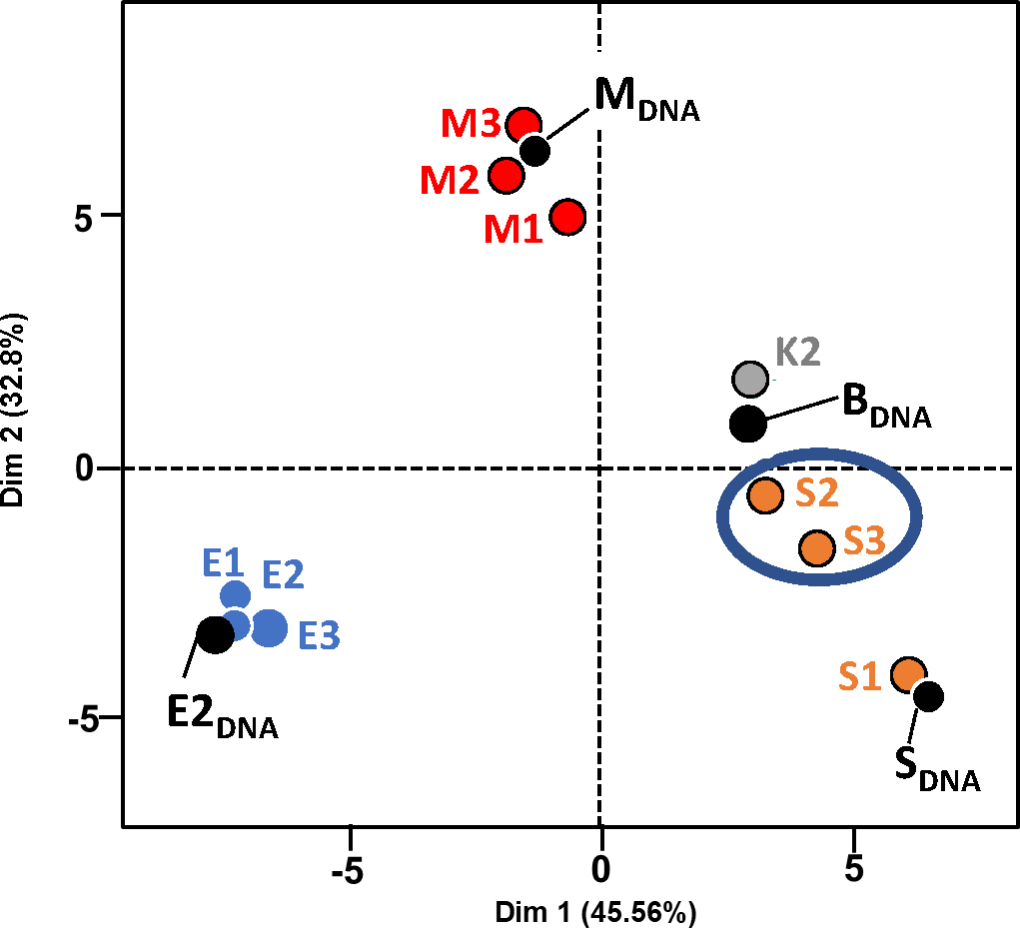
Principal component analysis (PCA) of CpGV isolates from ten RNA-Seq samples [M1–M3 (CpGV-M), S1–S3 (CpGV-S), E1–E3 (CpGV-E2), and mock infection sample K2] and respective viral DNA sequences based on the distribution and quantity of single-nucleotide polymorphisms. The relative positions of the DNAs of CpGV-E2, -M, -S, and -B (black dots) and the RNA-Seq samples (colored dots) are given in a two-dimensional factor map.

When a principal component analysis (PCA) was applied on the SNP frequency and SNP positions, the RNA-Seq samples M1–M3, E1–E3, and sample S1 clustered well with their respective DNA sequence data (Fig. 3). Sample K2 clustered with CpGV-B, whereas the samples S2 and S3 were placed between CpGV-M and CpGV-S, illustrating the mixed character of the two samples (Fig. 3).

SNP mapping in combination with PCA allowed the identification and quantification of viral genotype/isolate composition of every sample, as it is summarized in Table 2. Based on these results and to further compare the peroral infections with CpGV-M, CpGV-S and CpGV-E2, the RNA-Seq samples were combined into four treatment groups, i.e., M1–M3, S1, S2S3, and E1–E3 for further transcriptome analysis.

**TABLE 2:** Composition of RNA-Seq reads based on single-nucleotide polymorphism (SNP) mapping against viral DNA of CpGV-M, CpGV-S, and CpGV-E2

Treatments	CpGV-M			CpGV-S			CpGV-E2			H_2_O		
Replicate	M1	M2	M3	S1	S2	S3	E1	E2	E3	K1	K2	K3
Composition of RNA-Seq reads	100% CpGV-M			100% CpGV-S	70% CpGV-S 30% CpGV-M		100% CpGV-E2			–^*a*^	100% CpGV-B	–^*a*^

^*a*^ The RNA-Seq samples K1 and K3 of the mock infection (H_2_O) contained negligible amounts of viral reads.

### Gene expression patterns of different CpGV isolates in CpR5M

To examine the most highly expressed genes of the different CpGV isolates in CpR5M midguts, we compared the transcription levels of each gene within the different treatments. For normalization, the mapped reads of each viral gene were counted by the featureCounts package (Table S2) and the TPM (transcripts per million reads) value was calculated for each viral gene (Table S3).

**Fig 4:**
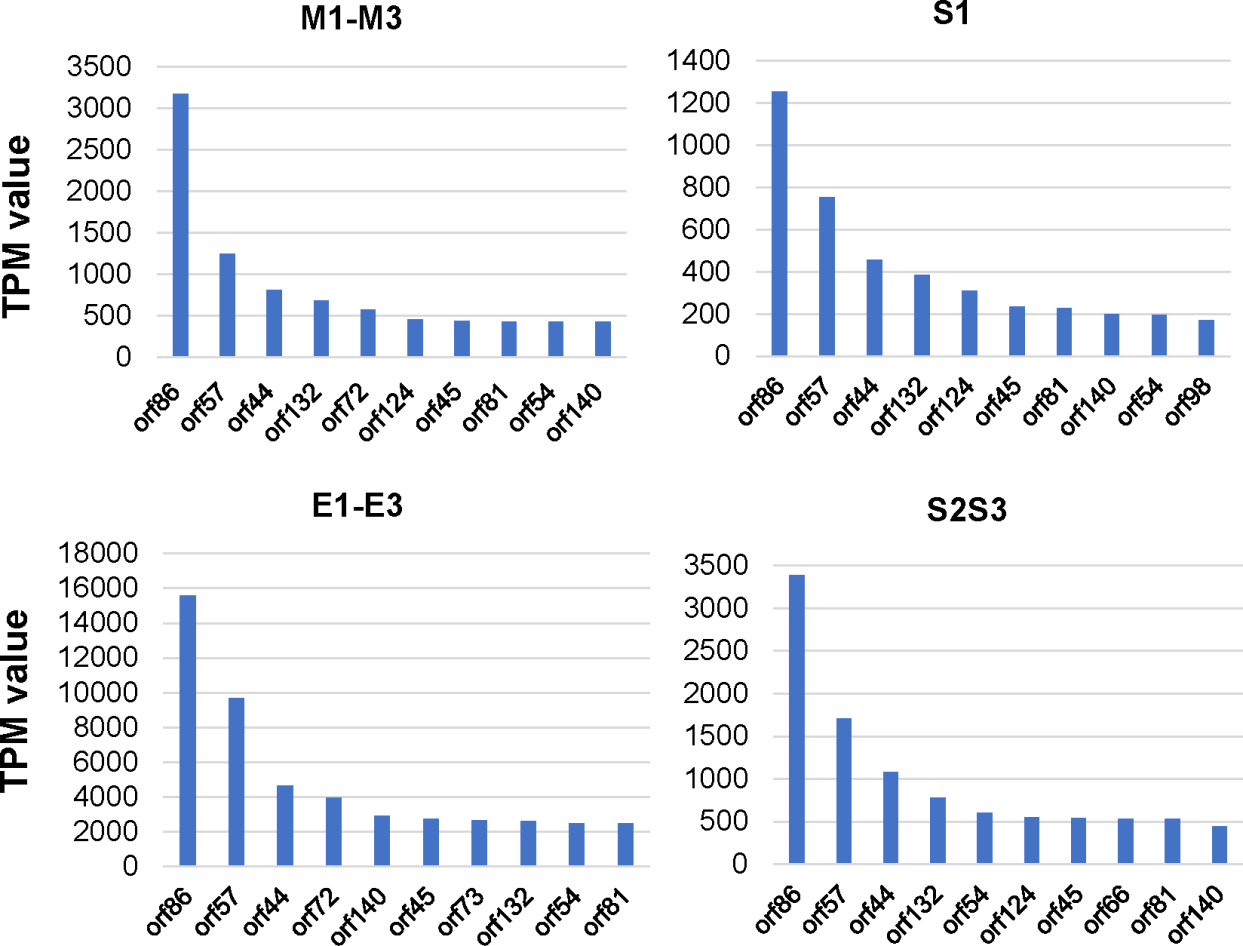
The most highly expressed open reading frames (orf) of CpGV in CpR5M midguts infected with CpGV-M (samples M1-M3), CpGV-S (S1, and S2S3), and CpGV- E2 (E1–E3) at 72 h post infection. Given are the 10 orfs with the highest normalized read counts (transcripts per million, TPM) ordered according to their TPM value.

The number of paired reads mapped to each CpGV gene and the number of total paired reads mapped of the samples were used to calculate the relative viral gene expression levels as TPM values, for each treatment (Table S3). The ten most highly expressed viral genes for each treatment were fundamentally the same for the three isolates (Fig. 4). The most abundant transcripts included the genes *orf86 (p6.9), orf57 (pp31*), and *orf44 (orf36L*). In most cases, the 4th to 10th most highly expressed genes showed rather similar expression levels, while the order of position was less stable (Fig. 4).

Because of the substantial difference in the viral gene expression level during the infection process of the resistance-prone isolates CpGV-M and CpGV-S and the resistance-breaking isolate CpGV-E2, direct comparisons of viral gene expression levels were difficult. Therefore, we used a combination of ranking and ratio methods to compare their expression in the different treatments and to identify gene candidates which we considered differently expressed in CpGV-M vs CpGV-E2 and CpGV-S vs CpGV-E2, respectively.

**Fig 5:**
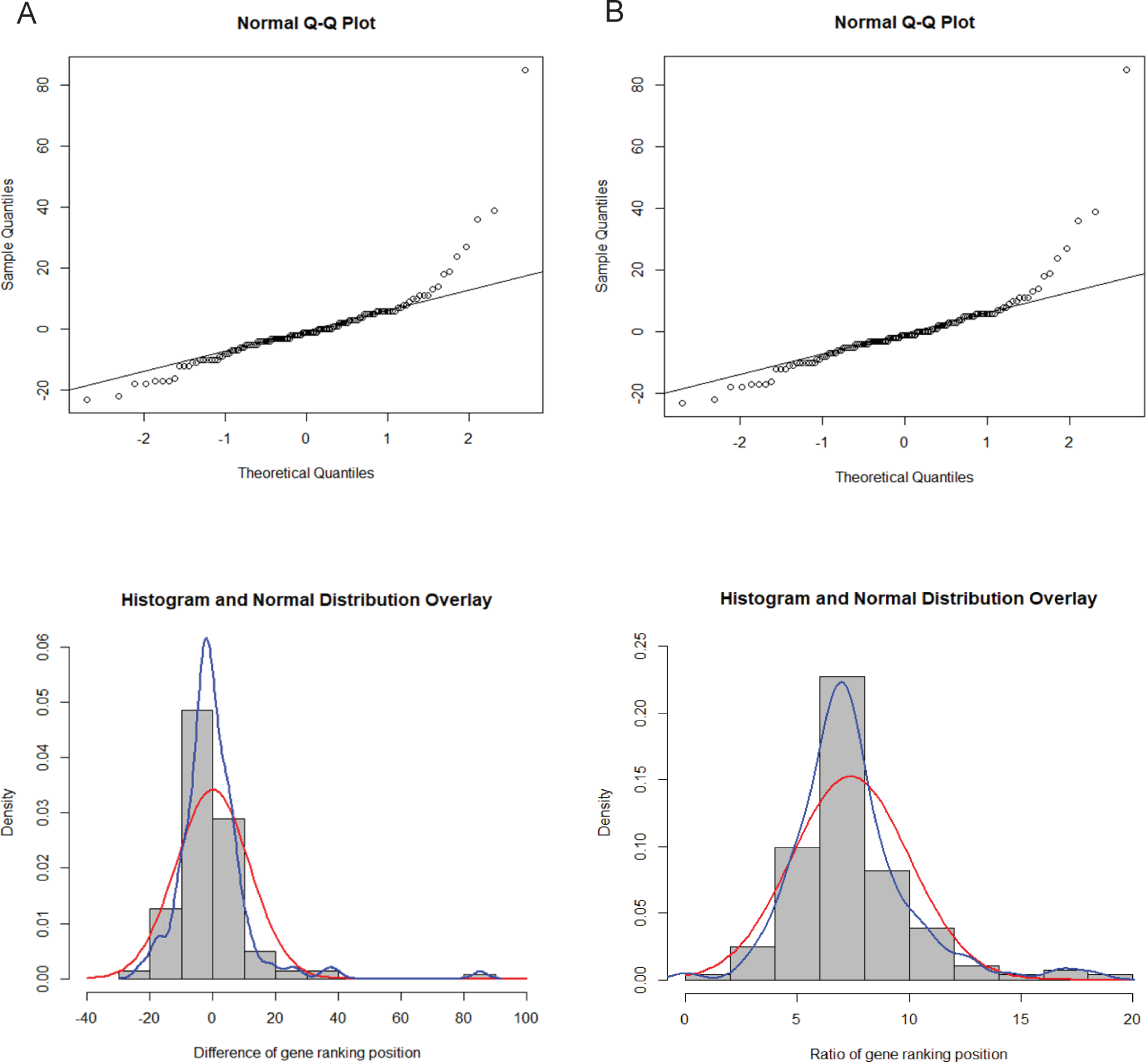
Normal distribution analysis with the quantile-quantile plots (Q-Q plots) (top) and the density graph (bottom) (**A**) for the data of ranking position change of CpGV-M vs CpGV-E2 and (**B**) for the data of ratio analysis of CpGV-M vs CpGV-E2.

By analyzing the distribution of the data set of gene ranking position differences between CpGV-E2 and CpGV-M using Q-Q plot and density graph, we found that the changes were normally distributed, and the mean value of this data set was around zero (Fig. 5A; Table S4). We considered those genes as differently expressed whose rank position change was located outside of the confidence interval of 90%. This threshold corresponded to genes whose ranking position changed more than +20 or less than −20 (Table S5).

**Fig 6:**
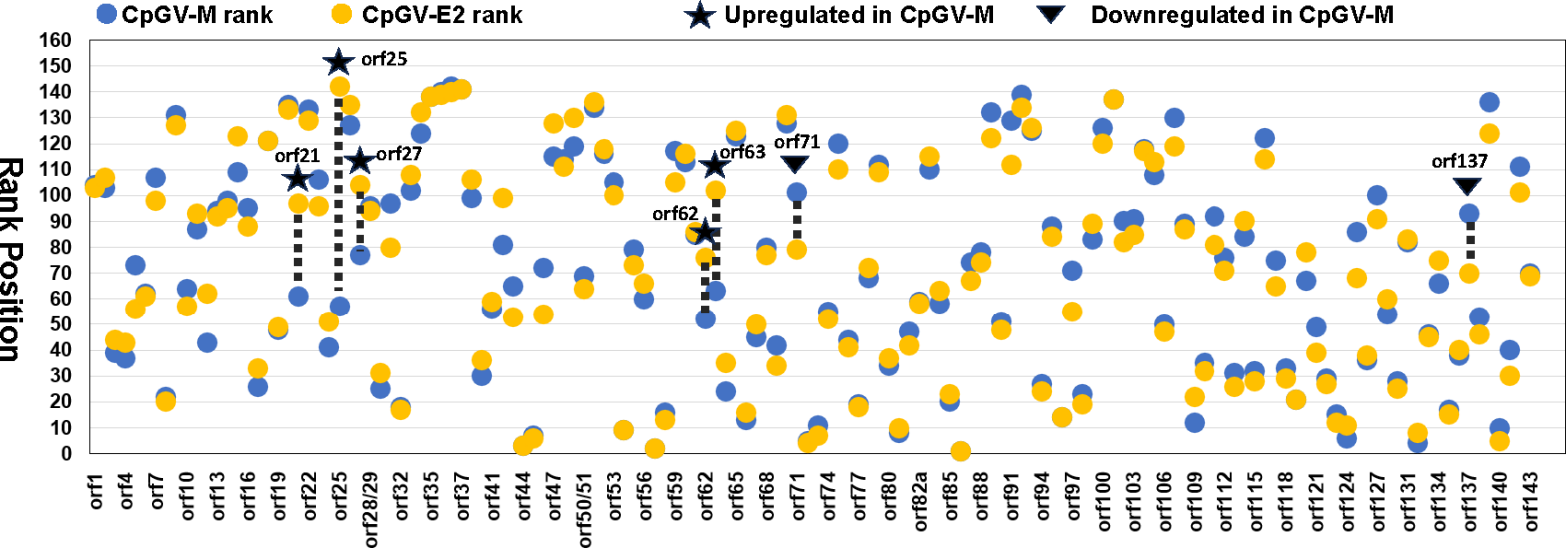
TPM ranking positions of each of the CpGV open reading frames (orfs) of infections with CpGV-M and CpGV-E2 at 72 h post infection. Based on TPM values, each orf was ranked in comparison to the expression of all other viral orfs of the same treatment. The ranking position of each orf expressed by CpGV-M (blue dots) was then compared with that of each orf expressed by CpGV-E2 (yellow dots). Selected orf names are given on the *x* axis. Black stars indicate orfs which rank positions are at least 20 positions higher in CpGV-M infections (upregulated), while black triangle indicate orfs with at least 20 rank positions lower in CpGV-M (downregulated) than in CpGV-E2.

In Fig. 5 and 6 , the statistical analysis and the gene rank position comparison between CpGV-M and CpGV-E2 are given for illustration. The same procedure was applied for the other treatment groups S1 and S2S3 to obtain the genes with substantial changes in the rank position (Table 3) (Fig. S2; Tables S4 and S5).

**TABLE 3:** Merged results on the different expression of genes of different groups using ranking method and ratio method^*a*^

ID	Gene	Promoter	Function	M		S1		S2S3	
				Rank	Ratio	Rank	Ratio	Rank	Ratio
*orf1*	*granulin*	l	struc	−1	7.11	15	7.39	**17**	2.80
*orf2*		e, l		4	6.24	3	11.34	5	4.07
*orf5*				−17	9.56	6	9.27	−5	5.19
*orf7*	*ie-1*	e	reg	−9	8.77	−2	10.41	−7	5.64
*orf9*	*ac145*	e, l	struc	−4	10.11	19	**5.60**	8	3.57
*orf11*	*cathepsin*	l	aux	6	5.80	20	7.10	**34**	2.11
*orf12*		l		19	3.84	**24**	**6.08**	**23**	2.34
*orf21*	*orf17L*			**36**	**2.87**	**34**	**4.79**	**28**	2.57
*orf23*	*pe/pp34*	e, l	struc	−10	9.28	**30**	**5.88**	−3	4.56
*orf25*		e		**85**	**0.05**	4	/*	9	**0.34**
*orf26*		l		8	4.03	**24**	**3.47**	1	3.35
*orf27*		e		**27**	3.30	**26**	**6.04**	**23**	2.51
*orf31*	*f-protein*	e	struc	−17	9.86	−23	20.80	−16	6.76
*orf34*		e		8	4.98	−6	/*	−5	8.09
*orf35*	*pif-3*	e, l	struc	0	8.66	12	**5.23**	6	2.16
*orf36b*		e		−1	16.48*	1	/*	1	4.31
*orf36a*		e		−2	/*	2	/*	−2	/*
*orf37*	*odv-e66*	l	struc	0	9.23*	4	23.87*	1	4.83
*orf42*	*orf35a*	e		18	4.52	**29**	**5.72**	6	3.70
*orf46*	*mp-nase*		struc	−18	10.22	−17	18.04	−21	8.11
*orf52b*	*ac106/107*	l		2	5.49	3	21.31*	1	3.23
*orf58*	*lef-11*		reg	−3	7.51	−15	23.73	−17	10.24
*orf59*	*sod*	l	aux	−12	12.52	−29	124.16*	−18	9.83
*orf61*		l		1	7.20	**24**	6.72	13	3.87
*orf62*		e, l		**24**	3.43	**58**	**1.76**	**28**	2.30
*orf63*	*bro*	e	reg	**39**	**2.61**	**23**	6.63	1	4.72
*orf64*		e, l		11	4.51	19	6.21	**18**	2.53
*orf65*		l		2	7.43	11	8.81	**18**	2.29
*orf67*		l		5	5.19	−27	21.93	−13	6.58
*orf69*		l		−8	9.36	1	12.63	6	4.42
*orf70*		l		3	6.35	−5	52.00*	−10	23.66
*orf71*	*p24capsid*	l	struc	−22	11.98	−16	16.35	−12	6.32
*orf74*	*lef-1*	e	reg	−3	7.65	−32	23.27	−10	5.91
*orf77*		e		−1	6.43	**−22**	30.75	−7	6.96
*orf89*	*pif-4*	l	struc	−10	12.80	−13	77.28*	−3	5.41
*orf91*	*odv-e25*	e, l	struc	−17	17.20	−7	17.30	−1	4.89
*orf92*	*p18*	l	struc	−5	14.50	3	15.63	−5	14.22
*orf95*	*lef-4*	l	reg	−4	7.51	−28	31.90	−8	5.78
*orf107*				−11	12.85	2	11.08	−9	6.72
*orf112*	*desmoplakin*	e	struc	−5	6.97	−10	13.55	−17	6.22
*orf114*	*pif-6*	e	struc	6	5.70	14	7.81	**18**	3.04
*orf115*		e		−4	7.34	−3	13.07	−10	6.20
*orf117*	*lef-9*	l	reg	−10	8.73	−20	19.35	−22	7.66
*orf121*				−10	8.75	−16	18.87	−7	6.09
*orf125*	*alk-exo*	e	aux	−18	10.34	−14	15.54	−14	6.42
*orf134*		l		9	5.03	**22**	6.30	5	4.53
*orf137*	*lef-10*	e, l	aux	−23	10.80	−2	11.50	−33	10.17
*orf139*		e		−12	18.23	−14	/*	−6	6.13
*orf141*	*egt*	e	aux	−10	9.57	−7	16.72	−11	7.64
*orf142*		e		−10	10.75	9	8.24	**35**	2.11
*orf143*				−1	6.77	20	6.69	**24**	2.42

^*a*^ Open reading frames (orfs) with numbers in bold font indicate upregulation and the numbers underlined mark downregulation compared to those of CpGV-E2 (samples E1−E3). For genes with asterisks "*", the expression value TPM in this group was below 1 (see Table S3), meaning no expression in the cell. M = CpGV-M (replicates 1–3), S1 = CpGV-S (replicate 1), S2S3 = CpGV-S (replicates 2 and 3).

Comparisons of the treatments CpGV-M vs CpGV-E2 showed that the ranking positions of most viral genes were very similar (see also Table S5). According to the ranking of TPM, five upregulated ORFs [*orf21* (*orf17L*), *orf25, orf27, orf62*, and *orf63* (*bro*)] and two downregulated ORFs [*orf71* (*p24 capsid*) and *orf137* (*lef-10*)] were identified for CpGV-M in comparison to the productive infection with CpGV-E2 (Fig. 6; Table 3). A comparable number of genes with substantial changes in their rank position were detected for S1 (16 genes) and S2S3 (17 genes). However, it is worth noting that only a small subset of the genes were consistent across the various treatments (Table 3).

**Fig 7:**
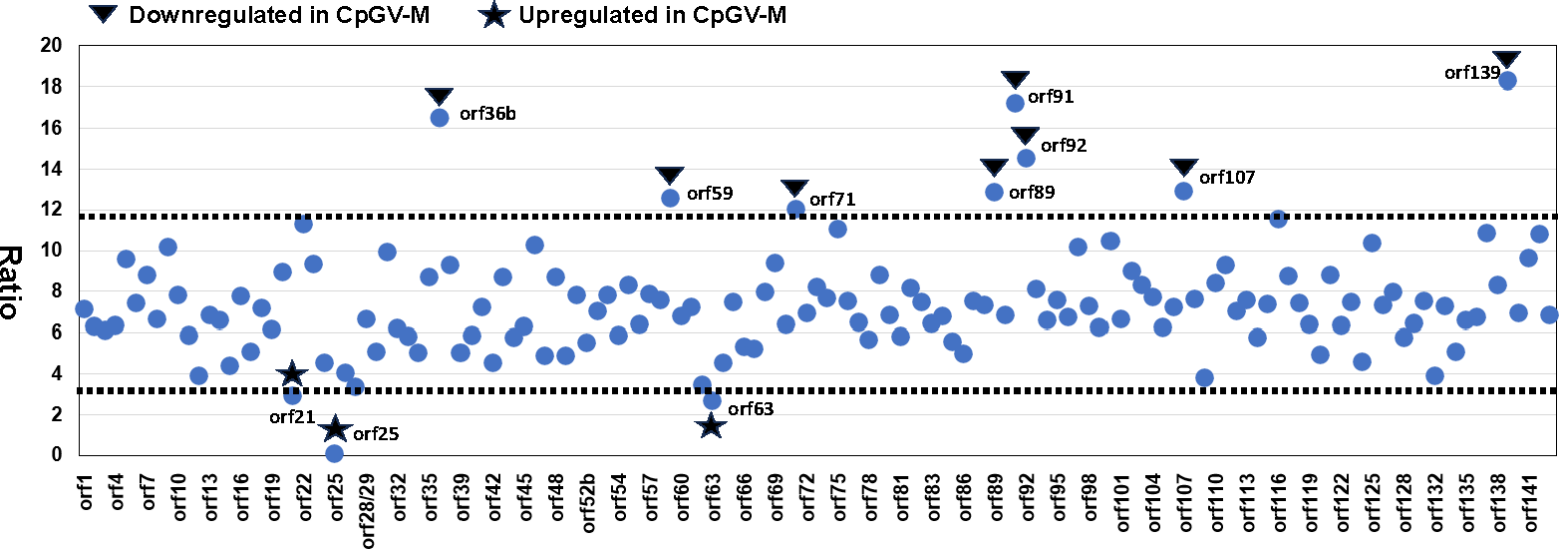
TPM ratio of each of the CpGV open reading frames (orfs) in infections with CpGV-M and CpGV-E2 at 72 h post infection. For each gene, the TPM value of CpGV-E2 was divided by that of the respective gene in CpGV-M. Orf names are indicated on the *x* axis. Black stars indicate orfs which are up regulated in CpGV-M (a ratio <3.08), while black triangle indicate orfs which are down regulated in CpGV-M (ratio >11.68) compared to CpGV-E2 (Table S2).

Using the ranking method alone to identify differences in gene expression between samples may be incomplete because some genes with small changes in the ranking position might show significant changes in the TPM value. Therefore, we combined the ranking method with the ratio method to make up for the shortcomings of the ranking method. Again, comparison of CpGV-M and CpGV-E2 is given as an example (Fig. 7). First, the TPM value of each gene of CpGV-E2 was divided by the TPM value from the same gene of CpGV-M to obtain the ratio of each gene between both treatments. The distribution analysis showed the ratio data set was also normally distributed (Fig. 5B), and then, the lower and upper 5% percentile of the ratio values were used to identify genes with substantial ratio difference (Table S6), predicting eleven downregulated and three upregulated genes of CpGV-M compared to CpGV-E2 (Fig. 7; Table S7). For the sample groups S1 and S2S3, twelve and five genes, respectively, were identified (Table S7).

Comparison of the ranking and ratio method further revealed that only a few genes were identified as differently expressed by both methods (Table 3). For example, for CpGV-M the ranking and ratio method identified seven and eleven genes, respectively, of which four genes were shared by both. A similar situation was found for the groups S1 and S2S3 (Table 3). Since we were interested in whether there was a common pattern of differences between the transcription of CpGV-M and CpGV-S vs the resistance-breaking CpGV-E2 that might not be fully reflected by the strict conditions of the two methods, we performed a rating of the information provided by the two methods and considered those genes as differently expressed when they were supported by the majority of samples and methods (Table 3). Following this procedure, we identified four genes with ranking method and/or ratio method, and for which relative expression levels were significant lower in infections with CpGV-M and CpGV-S than with CpGV-E2*: orf59* (*sod*), *orf89* (*pif-6*)*, orf92* (*p18*), and *orf137* (*lef-10*) (Table 3, underlined font). Furthermore, we identified five genes with higher relative expression levels in the CpGV-M than in the CpGV-E2 at 72 hpi. These genes included *orf21* (*orf17L*)*, orf25, orf27, orf62,* and *orf63* (*bro*) (Table 3, bold font). None of the highly expressed genes identified before (Fig. 4) belonged to these genes.

## DISCUSSION

Since the type I resistance of codling moth to CpGV-M was discovered (15), numerous studies revealed in-depth insight into (i) its resistance inheritance and mechanism of this resistance (17, 18), (ii) overcoming resistance by other CpGV isolates (14, 18, 24, 30, 31), and (iii) the transcription of CpGV infecting codling moth larvae (19, 20). Recently, the type II of CpGV resistance with a different mode of inheritance and different susceptibility to CpGV isolates was reported (22, 25). To further elucidate the nature of type II resistance, we performed comparative transcriptional analyses with CpGV-M and CpGV-S, which both are prone to type II resistance, and the resistance-breaking isolate CpGV-E2 (26). To compare transcription of the three viruses, a single time point, 72 hpi, was chosen because previous studies had demonstrated that during *in vivo* infection of CM larvae the onset of virus transcription in the midgut starts between 48 and 72 hpi (19, 20). By covering the transcription at 72 hpi, significant differences in early events of infection should be notable.

We performed high-throughput sequencing of RNA samples and mapped the obtained sequencing reads to the genome of CpGV-M reference genome. Then, these read mappings to the viral genes were counted. We found that the proportion of viral reads to total reads in samples infected with CpGV-M (0.35%) and CpGV-S (0.42%) was much lower compared with those infected with CpGV-E2 (2.90%). The observation of CpGV-M and CpGV-S transcripts, though at low level, indicated that both viruses were able to enter midgut cells and initiate transcription there. In a recent study, the proportion of viral reads to total reads in susceptible CpS larvae infected with CpGV-M was 1.3% at 48 hpi, and 11.9% at 96 hpi (19). The proportion of viral reads of the resistance-breaking CpGV-E2 in CpR5M larvae was in the same range of that of the permissive infection of CpGV-M in CpS larvae, suggesting a similar transcription activity of CpGV-E2 in CpR5M and CpGV-M in CpS larvae, and thus a similar permissive infection of CpGV-E2 in the resistant CpR5M larvae. In a RNA-Seq study of AcMNPV, the average percentage of viral reads in the midgut of infected larvae of *Trichoplusia ni* ranged from 0.003% (0 hpi) to 27.9% (72 hpi) (32). Compared with AcMNPV, the proportion of reads of the permissive infection with CpGV-E2 at 72 hpi is 10-fold lower and might reflect differences in the experimental set-up, viral infectivity, or host-virus interaction.

Interestingly, one of the three untreated control replicates (K2) contained a significant proportion of CpGV-specific transcripts, indicating that this sample was contaminated with a CpGV infection. Since activation of covert baculovirus infections is not uncommon in *in vivo* infection experiments (27), we further analyzed sample K2 as well as all other treatment samples for the identity of the infective agent. This aim was achieved by SNP mapping of the transcripts using the bacsnp tool (29) and was based on the known SNP patterns of the virus isolates used in this study (10, 14). Indeed, it was found that sample K2 originated from infection with CpGV-B isolate, which is a novel type of CpGV isolate found in the rearing of the host strain CpR5M (unpublished). In addition, two of three replicates (S2 and S3) of the infection with CpGV-S contained mixed infections with about 70% and 30% transcripts typical for infection with CpGV-S and -M, respectively. Since the infection experiments were performed independently, it can be ruled out that contaminant presence of CpGV-B in K2 and CpGV-M in the treatment with CpGV-S originated from external contamination during handling of larvae rather than by activation of covert viruses present in the test insects. The presence of such covert viruses and their transcripts in reared insect is not unusual, as recently noted for CpS larvae (19) or when S*podoptera frugiperda* and *Trichoplusia ni* larvae were analyzed for AcMNPV transcripts (33). As infection by a second pathogen represents a major threat to host survival, the activation of overt baculovirus disease reflects a switch to a horizontal transmission strategy, as competition between viruses favors increased virulence (34–37).

Owing to of the possibility of virus contamination, either through activation of covert viruses or by laboratory contamination, it is of utmost importance to identify the infective agent in the transcripts when performing *in vivo* infection experiments. Such contaminant or activated viruses may escape conventional quality control done by PCR, RT-PCR, qPCR, or similar, especially when the contaminating virus is highly identical to the inoculum. The SNP analyses on the transcripts, as performed in this study, allow an unequivocal *a posteriori* identification of the infective agent in the samples. This method can be used not only to analyze RNA-Seq samples of baculovirus, but also to detect other viruses (38). Principal component analysis of the samples’ SNP features revealed different clusters which were used for gene expression analysis.

As the aim of this study was to compare viral gene expression of different viruses in CpR5M larvae, the TPM value was used for normalization of raw reads for the gene expression analysis. TPM values for viral genes were calculated using the total number of viral paired reads and host paired reads; therefore, expression values (TPM) were comparable to those for other viral genes examined not only in the same sample (group) but also across samples (groups) (39). The normalization method is of crucial importance because different normalization methods lead to significantly different results (40). The common normalization methods have the following three classes: (i) TPM (transcript per million reads), (ii) RPKM/FPKM (reads/fragments per kilobase of exon per million reads/fragments mapped), and (iii) DESeq2’s median of ratios/EdgeR’s trimmed mean of M values (TMM) (41). The RPKM/FPKM method could not be applied because the total number of RPKM/FPKM normalized counts for each sample is different and can, therefore, not be used to compare the normalized counts for each gene equally between samples (40, 41). DESeq2’s median of ratios/TMM makes the assumption that the majority of genes are not differentially expressed, the majority of genes in each sample should have similar ratios within the sample (41). This assumption, however, is not valid for viral genes differently expressed between a resistance-breaking virus and a virus not breaking resistance. To overcome the limitations of these normalization methods, analyses of TPM values in combination with ranking (and ratio) method were applied to compare gene expression of viral genes between samples (19, 32, 40).

The 10 most highly expressed genes were similar in all groups independently, whether a resistance-prone or resistance-breaking CpGV isolate was used as inoculum. These most highly expressed genes included *orf86* (*p6.9*)*, orf57* (*pp31/39K*)*, orf44* (*orf36L*)*, orf132, orf45, orf54,* and *orf140* (*fgf-3*). These results corroborate previous studies using the CM strains CpS (susceptible) and CpRR1 (type I resistance) which identified similar highly expressed genes at 48 and 72 hpi (19, 20). In *T. ni* infected by AcMNPV at 72 hpi, the most highly expressed genes were *polh*, *p6.9, Ac-orf76, p10, odv-ec27, Ac-bro, Ac-orf75, odv-e25, Ac-orf74, odv-e18, ctx, Ac-orf82, pp31, Ac-orf5,* and *cg30 (32*). Thus, only *p6.9* and *pp31* were highly expressed in both CpGV and AcMNPV infection.

Type II resistance to the CpGV-M and CpGV-S inhibits a systemic viral infection although systemic resistance against CpGV-S could be circumvented by injecting budded viruses into the hemocoel (26). Therefore, two different mechanisms or at least some variation in the resistance mechanisms was proposed for CpR5M when infected with CpGV-M and CpGV-S (26). However, no substantial difference was found in the transcription between CpGV-M and CpGV-S in the midgut. This suggests that the blockage of CpGV-S infection does not only occur at the cell entry but also during the infection of midgut cells similar to the assumption made for CpGV-M.

As a result of the combination of the ranking and ratio method, we identified nine genes for CpGV-M and -S which were either upregulated or downregulated. Notably, the majority of upregulated genes belong to early transcribed genes, whereas most of the down-regulated genes were late genes. These results indicate that transcription of CpGV-M and -S is significantly lower and delayed compared to resistance-breaking CpGV-E2. Due to the compromised onset of viral gene transcription in CpR5M midguts for both CpGV-M and CpGV-S, the transcription of late viral genes is further reduced. A similar situation was noted for the infection of CpGV-M in type I-resistant CM larvae (20), which may hint to a similar but differently expressed resistance mechanism in type I- and type II-resistant codling moth.

In summary, the following conclusions can be drawn from our study: (i) SNP distribution in the RNA-Seq data can be used for *a posteriori* identification of the infective agent. (ii) Identification of infective agent is crucial, esp. in *in vivo* infection experiments, when activation of a covert virus infection is a possibility. (iii) No substantial difference between CpGV-M and CpGV-S transcription was found in CpR5M although a midgut-related resistance factor was proposed for CpGV-S. This indicates that CpGV-S can enter midgut cells, and midgut cell limit one or more steps of virus transcription or replication. (iv) The observed transcription profiles indicate similar transcription patterns but highly reduced transcription levels for CpGV-M and CpGV-S compared to CpGV-E2 proposing an early block of virus replication in type II resistant CM.

## MATERIALS AND METHODS

### Viruses and insects

Three different isolates of Cydia pomonella granulovirus (CpGV) (species *Betabaculovirus cypomonellae*, family *Baculoviridae*, order *Lefavirales*), i.e., CpGV-M (belonging to genome group A), CpGV-S (genome group E), and CpGV-E2 (genome group B), were used (1, 7, 42). Stocks of occlusion bodies (OBs) of CpGV-M, -S, and -E2 were stored at −20°C; their identity and purity were determined by whole-genome sequencing (14) before use. OB concentration was determined using a Petroff-Hausser counting chamber (depth 0.02 mm) in dark field microscopy (Leica DMRBE) (43).

Two codling moth (*C. pomonella*) strains were used: Strain CpR5M derived from the field population NRW-WE, which had been selected on CpGV-M for five generations; CpR5M is resistant to CpGV-M and -S but not to CpGV-E2, whereas strain CpS is fully susceptible to all three virus isolates (25). All CM larvae were reared under laboratory conditions at 26°C with 16/8 h light/dark photoperiod and about 60% relative humidity on a modified diet described previously (44). For rearing details, see Bathon (45).

### Infection of CpR5M larvae with different CpGV isolates

Third instars of CpR5M and CpS were infected with CpGV-M, -S, or -E2 via oral administration of viral OBs. Infection was performed as described by Wennmann et al. (20). In brief, larvae were starved overnight before each larva was offered a small cube (~1 mm^3^) applied with 10^4^ viral OBs. Mock-infected control larvae were provided diet cubes without virus OBs. Only larvae which had ingested the provided piece of diet overnight were included in the experiment and transferred to virus-free diet. This time point was set as the experimental starting point (0 hpi). At 72 hpi, a randomly selected subset of larvae was collected and stored individually in 1.5 mL centrifuge tubes at −80°C for later isolation of midguts and RNA extraction. The remaining larvae were reared further to determine larval mortality after 7 days post infection (dpi) and 14 dpi. The infection experiment was repeated independently three times. Mortality data were tested for significance using Tukey Honest Significant Differences (HSD).

### Midgut dissection and RNA sequencing

To extract the total RNA from midguts, the larvae were thawed and immediately dissected on a wax dish under a binocular. After removal, the midguts were washed to remove intestinal debris. For each replicate of the four infection experiments (CpGV-M, CpGV-S, CpGV-E2, and mock infection), twelve midguts were pooled for RNA extraction. The twelve midguts of each replicate were pooled in a 1.5-mL Eppendorf tube with 250 µL LBA and TG buffer of the RNA isolation kit (ReliaPrep RNA Tissue Miniprep System, Promega), followed by RNA isolation according to the manufacturer’s instruction. RNA quality was determined using Agilent Bioanalyzer 2100 to check for RNA degradation. After quality and quantity check, 2 µg of total RNA were sent for RNA sequencing (StarSEQ GmbH, Mainz, Germany) on an Illumina NextSeq 500 sequencer. Starting from total RNA, the mRNA was purified for library preparation using TrueSeq stranded dual index RNA library preparation kit. Each replicate was sequenced with an estimated output of 50–60 million reads (paired end, 150 nt in length).

### Read processing

Raw RNA-Seq reads were ﬁrst processed to trim adapter and low-quality bases using Trim Galore v0.6.5 (https://github.com/FelixKrueger/TrimGalore). Trimmed reads shorter than 40 bases were discarded. To analyze the viral transcriptome from different viral isolates, the sequences of the CpGV-M genome (GenBank accession no. KM217575) and the codling moth genome (46) (http://www.insect-genome.com/cydia/download.php) were merged into one genome reference file to be used for mapping. Finally, the cleaned reads from each replicate sample were mapped to the merged genome reference file using HISAT2 (47), allowing two mismatches per 150 bp read.

Following alignments, the number of mapped reads to each of the 143 viral genes and 17,184 host genes was counted using the featureCounts software of the Subread package to get a raw count of reads (Table S2) (48). Raw read counts were then normalized to transcripts per million mapped reads (TPM) using excel software as described previously (40). Note: To examine expression of viral genes relative to total host expression, we calculated TPM values (Table S3) by dividing the number of mapped reads by the total number of mapped reads (host plus viral reads) instead of the total number of mapped viral reads only.

### Coverage plot of RNA-Seq data

To evaluate whether the whole viral genome of CpGV-M, -S, and -E2 was transcribed in CpR5M midguts, coverage plots of each of the three replicates of CpGV-M, -S, and -E2 infections plus the three replicates of the mock-infected control were generated by mapping the reads to the genome sequence of CpGV-M, using BWA-MEM mapper on Galaxy server of the Julius Kühn-Institut and counting every position of reference using the Galaxy plugin Mpileup (29). After Mpileup was run on mapped reads, the output Variant Call Format (VCF) file contained the read count for each reference nucleotide. The obtained VCF file used for the coverage plots contained the raw VCF output of Mpileup including variant and non-variant sites. The coverage plots were visualized using R studio (R version 3.6.3 in RStudio 1.4.1717).

### Distinguishing and quantifying viral genotype in RNA sequencing samples

To distinguish and quantify the CpGV genotype represented in the individual RNA-Seq samples, we developed a novel mapping approach based on a previous method published by (29). The complete workflow is depicted in Fig. S3. The trimmed and filtered reads were mapped to the reference sequence of CpGV-M (see above) and CpGV-E2 (GenBank accession no. KM217577) using BWA-MEM v0.8.0 (49) to get two Sequence Alignment Map (SAM) files for both viruses. Then, the SAM files were transformed into Binary Alignment Map (BAM) files, and all unmapped reads were removed from BAM files using SAM tool package (50) to reduce the size of BAM files.

The BAM files of all samples were used to call all variant positions in correlation to CpGV-M and CpGV-E2 using MPileup V2.1.1 to get two individual sets of output files for analysis (29, 50). The MPileup parameters were the following: (i) the number of high-quality bases (DP) and number of high-quality bases for each observed allele (DRP) were included; (ii) insertions and deletions (indels) were not included. Then, those non-variant positions were filtered using BCFTools V1.0 (50) to get VCF files containing total read depth of each SNP position, the count of reference, and the three possible alternative nucleotides. Since two different virus isolates were used as reference, two sets of output VCF files were obtained for the following analysis.

In previous publications, the CpGV genotype(s) could be identified using genotype-specific SNPs (14). The filtered SNP frequency and position were used to identify the CpGV genotypes expressed in the RNA-Seq samples (51, 52).

After identification of the viral genotype represented in the RNA-Seq data, the detailed quantification within RNA-Seq samples was performed using the R package bacsnp v0.1.0 (29). First, the absolute counts of reference and alternative nucleotides were transformed to relative frequencies. Then, the SNP table was filtered to reduce the noise of sequencing and assembling errors using the following parameters: (i) only SNP positions at locations with an absolute total read depth over 20 were included; (ii) alternative read counts were higher than 10; (iii) the relative frequency (ƒ) of reference and alternative nucleotides exceeded 0.05 (29). Then, the specificity of each variant position based on the alternative nucleotide was defined and determined using the *de novo* SNP calling method from bacsnp package in RStudio. Finally, tthe SNP positions and their frequencies was visualized in R. We performed cluster analysis on normalized read counts to examine the similarity of RNA-Seq data with the respective CpGV genomes. Hierarchical clustering was performed using the Euclidean distance metric on the log2-transformed TPM values using R software (39, 53).

### Expression proﬁling and differential gene expression

The most highly expressed CpGV genes were identified based on the calculated TPM values. Owing to the fact that absolute expression levels vary considerably among different CpGV isolates in the midgut of CpR5M larvae and since we aimed to compare the expression levels in the overall context of the virus program of gene expression, TPM ranking was used for expression proﬁling (19). First, the TPM value of each viral gene was ranked for different treatment groups. Then, the rank change was determined by subtracting the respective rank from the rank of the same gene of CpGV-E2 according to the following formula: rank change = rank of orf[*i*] of TPM(E1–E3) – rank of orf[*i*] of TPM[*X*], with *i* = 1–143 and *X* = M1–M3, S1, S2S3. The ranking method might miss those different expression genes whose ranking position does not change significantly, but whose expression value changes significantly (19, 32). As an alternative method to compare the CpGV gene expression, a ratio (multiple change)-based expression proﬁling was employed. To calculate the multiple change of gene expression among the different CpGV isolates, the TPM value of each gene of CpGV-E2 infection was divided by the TPM values for same gene of CpGV-M and CpGV-S, according to ratio = TPM(E1–E3)orf[*i*]/TPM[X]orf[*i*], with *i* = 1–143 and *X* = M1–M3, S1, S2S3. After analyzing the normal distribution of the ranks and ratios, the 90% percentiles were calculated for each analyzed experimental group, and all genes with ranks or ratios belonging to the upper or lower 5% percentile were considered significantly differently expressed.

## Data Availability

The project data, including all raw sequencing data (SRA), are available under NCBI BioProject PRJNA924089 (accession no. SRR23086231 to SRR23086245).

## SUPPLEMENTAL MATERIAL

Fig. S1 jvi.00537-24-s0001.pdf Sequencing coverage plots.

Fig. S2 and S3 jvi.00537-24-s0002.pdf Normal distribution analysis and bioinformatic workflow.

Table S1 jvi.00537-24-s0003.docx Viral reads in untreated controls.

Table S2 jvi.00537-24-s0004.docx Raw paired reads counts of viral genes.

Table S3 jvi.00537-24-s0005.docx TPM value of every gene.

Table S4 jvi.00537-24-s0006.docx Statistical analysis of ranking position.

Table S5 jvi.00537-24-s0007.docx Change of rank position.

Table S6 jvi.00537-24-s0008.docx Statistical analysis of TPM ratio.

Table S7 jvi.00537-24-s0009.docx Ratio of TPM value compared with CpGV-E2.

ASM does not own the copyrights to Supplemental Material that may be linked to, or accessed through, an article. The authors have granted ASM a non-exclusive, world-wide license to publish the Supplemental Material files. Please contact the corresponding author directly for reuse.
