# Supplementary material for: Transcriptome analysis of CpGV in midguts of type II resistant codling moth larvae and identification of contaminant infections by SNP mapping of RNA-Seq data
Source: J Virol. 2024 Jun 27;98(7):e00537-24. doi: 10.1128/jvi.00537-24 (PMC11265400; doi:10.1128/jvi.00537-24)
Supplement: Fig. S1 — Sequencing coverage plots. [file jvi.00537-24-s0001.pdf]

FIG S1 Coverage plots of viral RNA-Seq reads mapped to the annotated CpGV-M genome sequence (KM217575). All three replicates of three virus treatments with CpGV-M (M1-M3), and the mock infection control (K2) (below) and CpGV-S (S1-S3), CpGV-E2 (E1-E3) (next page) are shown. The controls K1 and K3 are not given as they contained neglectable numbers of CpGV reads. Regions with zero coverage are shaded red. Repeat regions are in grey color. Vertical axis stands for read counts, and horizontal axis stands for reference position. Note the different scale of the y axes.

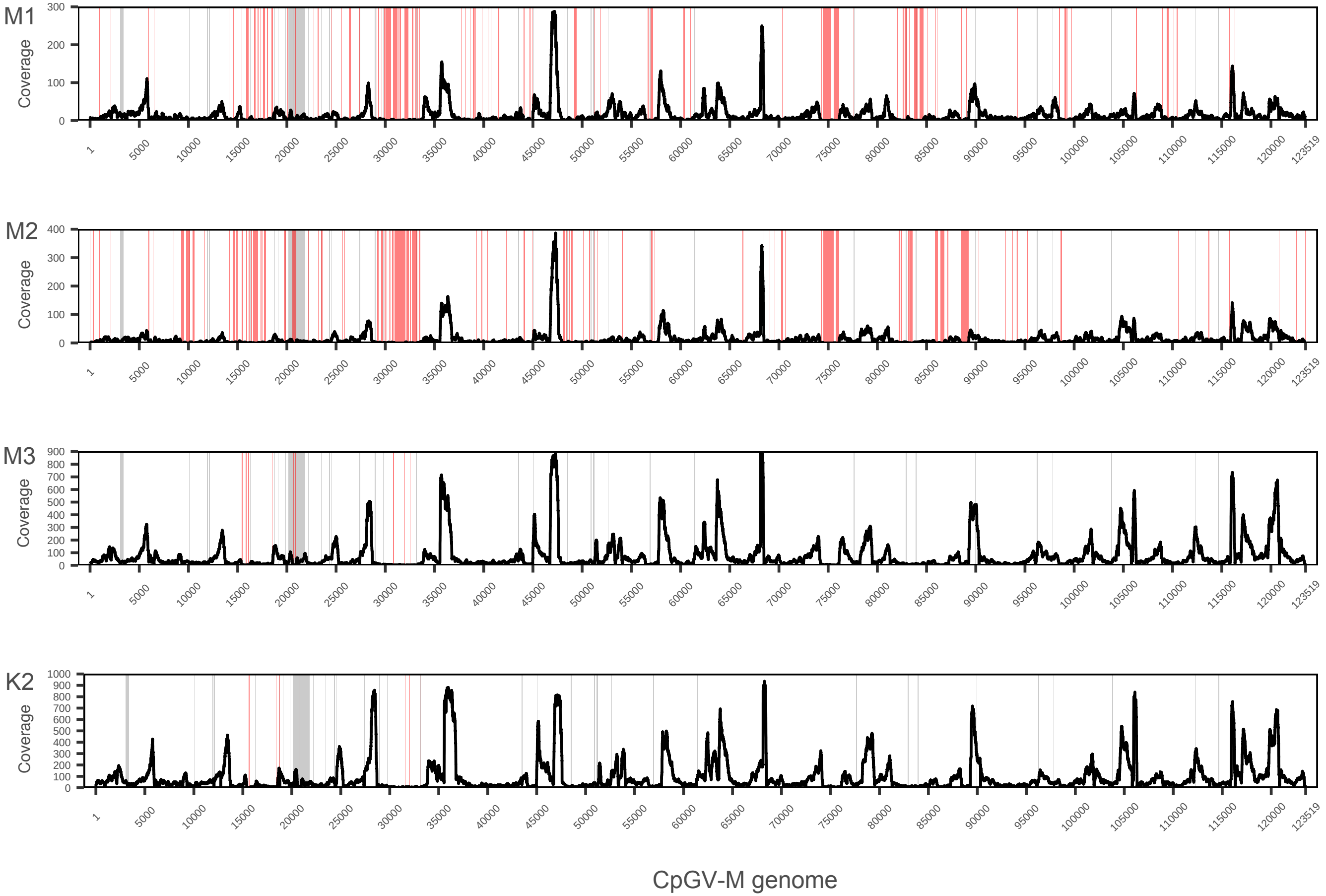

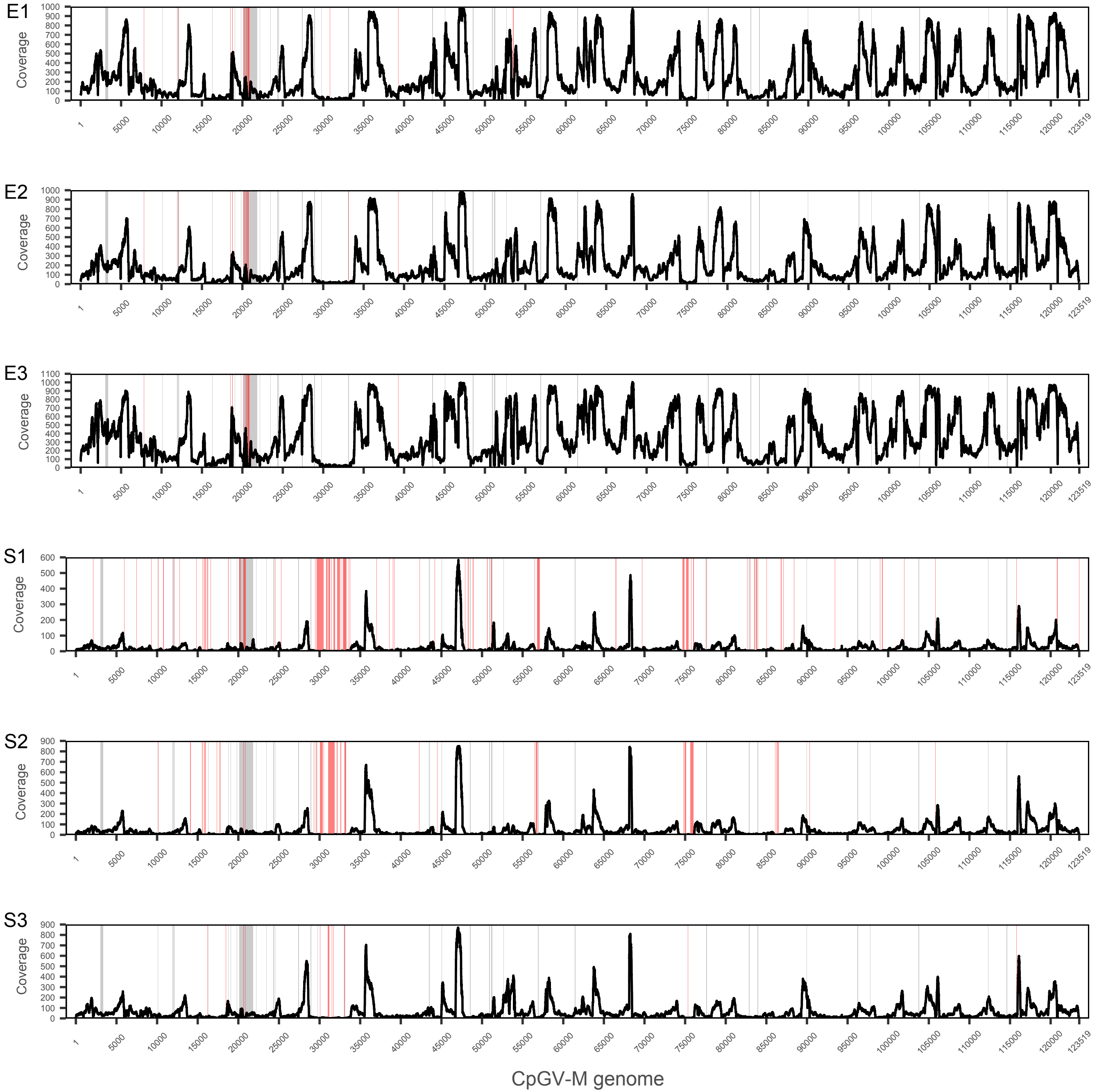
E1
E2
E3
S1
S2
S3
Coverage
1
5000
10000
15000
20000
25000
30000
35000
40000
45000
50000
55000
60000
65000
70000
75000
80000
85000
90000
95000
100000
105000
110000
115000
120000
123519
CpGV-M genome
