# Supplementary material for: Transcriptome analysis of CpGV in midguts of type II resistant codling moth larvae and identification of contaminant infections by SNP mapping of RNA-Seq data
Source: J Virol. 2024 Jun 27;98(7):e00537-24. doi: 10.1128/jvi.00537-24 (PMC11265400; doi:10.1128/jvi.00537-24)
Supplement: Fig. S2 and S3 — Normal distribution analysis and bioinformatic workflow. [file jvi.00537-24-s0002.pdf]

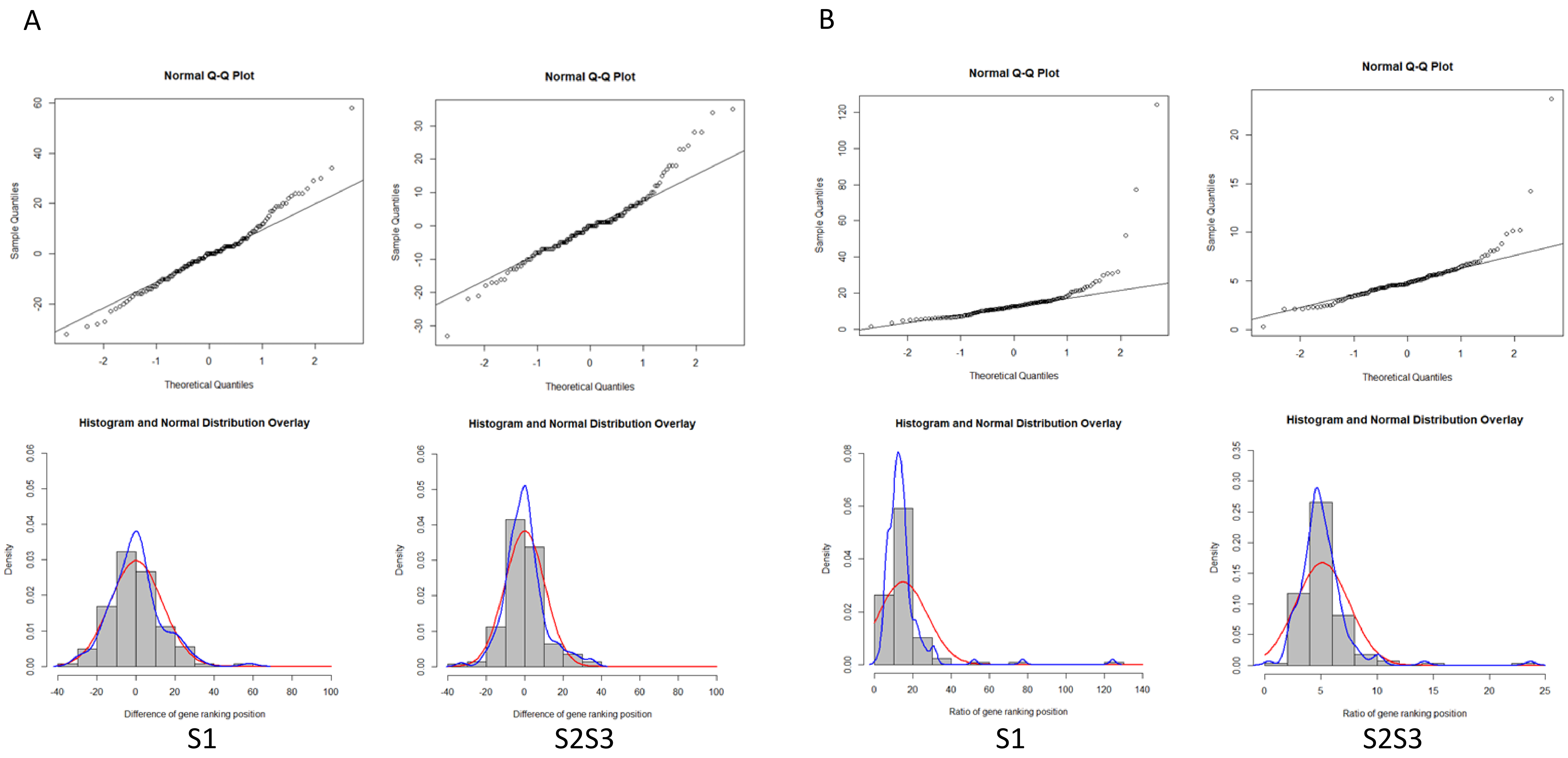

FIG S2 Normal distribution analysis with the quantile-quantile plots (Q-Q plots) (top) and the density graph (bottom) (**A**) for the data of ranking position change of the samples S1 and S2/S3 vs. CpGV-E, and (**B**) for the data of ratio analysis of S1 and S2S3 vs. CpGV-E2.

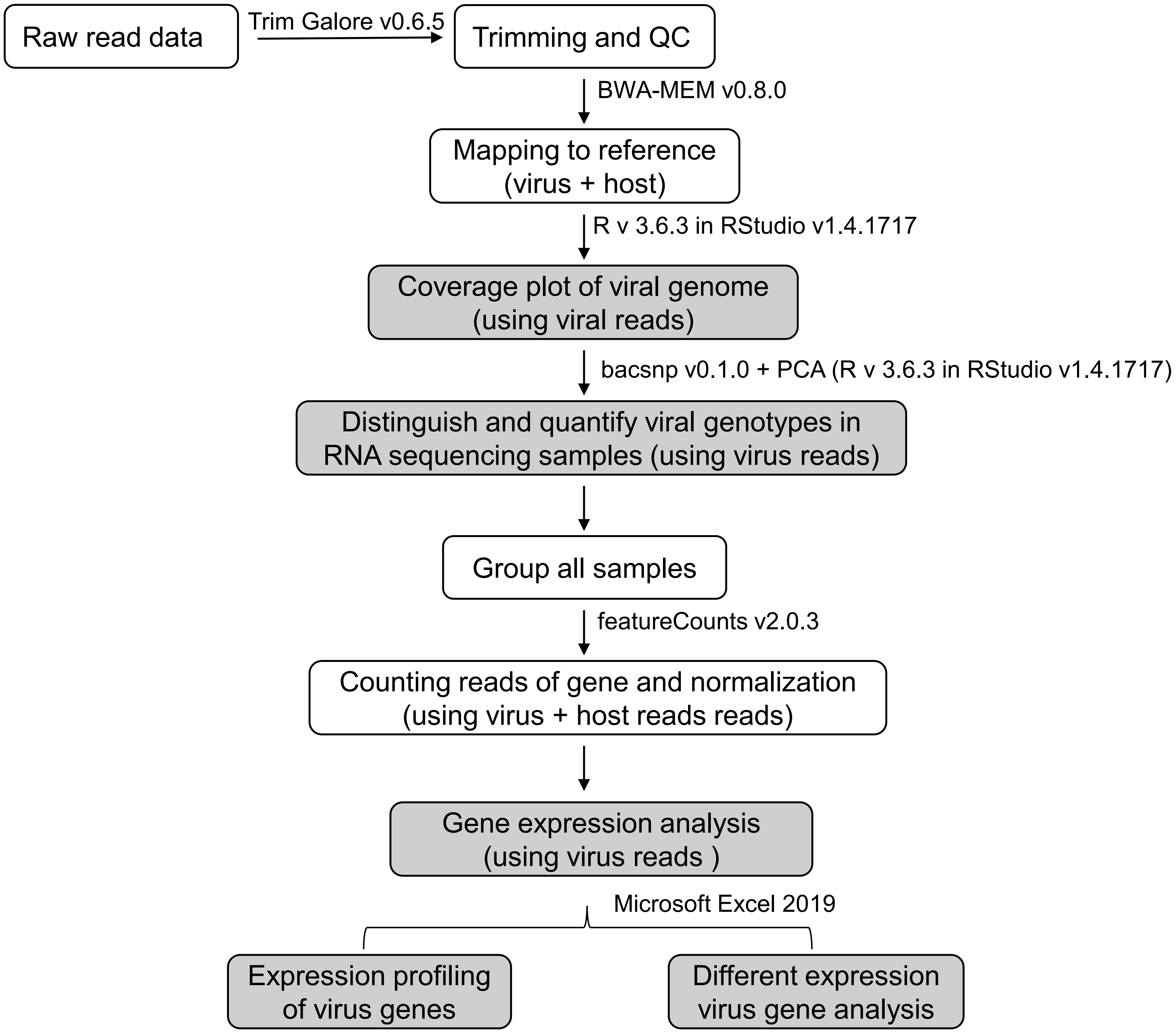

FIG S3 Work flow from raw data output of Illumina sequencing to downstream RNA-Seq analysis. White boxes stand for the data and data processing steps, whereas the grey boxes indicate the analysis steps. For details see Materials and Methods.
