# Supplementary material for: Transcriptome analysis of CpGV in midguts of type II resistant codling moth larvae and identification of contaminant infections by SNP mapping of RNA-Seq data
Source: J Virol. 2024 Jun 27;98(7):e00537-24. doi: 10.1128/jvi.00537-24 (PMC11265400; doi:10.1128/jvi.00537-24)
Supplement: Table S1 — Viral reads in untreated controls. [file jvi.00537-24-s0003.docx]

| **Orf** | **Gene** | **K1** | **K3** |
| --- | --- | --- | --- |
| orf17 | *iap-3* | - | 2 |
| orf30 | *-* | 2 | - |
| **orf44** | ***orf36L*** | **2** | **4** |
| orf55 | *odv-ec43* | 3 | - |
| **orf57** | ***pp31/39K*** | **5** | **1** |
| **orf72** | ***-*** | **3** | **5** |
| orf78 | *-* | - | 1 |
| orf81 | *dbp* | - | 4 |
| **orf90** | ***helicase*** | **4** | **6** |
| orf95 | *lef-4* | - | 2 |
| orf113 | *lef-3* | 2 | - |
| orf118 | *fp25k* | - | 2 |
| orf120 | *DNA ligase* | - | 2 |
| orf121 | *-* | - | 1 |
| orf124 | *-* | - | 9 |
| orf126 | *helicase-2* | 2 | - |
| Total |  | 23 | 39 |

**TABLE S1** Read counts in the RNA-Seq samples K1 (replicate 1) and K3 (replicate 3) of the mock infection control. Bold letters indicate those open reading frames (orfs), which reads were found in both K1 and K3.
