# Supplementary material for: Transcriptome analysis of CpGV in midguts of type II resistant codling moth larvae and identification of contaminant infections by SNP mapping of RNA-Seq data
Source: J Virol. 2024 Jun 27;98(7):e00537-24. doi: 10.1128/jvi.00537-24 (PMC11265400; doi:10.1128/jvi.00537-24)
Supplement: Table S2 — Raw paired reads counts of viral genes. [file jvi.00537-24-s0004.docx]

**TABLE S2** Raw counts of paired read of viral genes for all RNA-Seq samples of CpGV-M (M1-M3), CpGV-S (S1-S3), CpGV-E2 (E1-E3) and sample K2 (mock infection control) determined by featueCounts software. Given are the start, end, orientation relative to orf1, and the length of the open reading frames (orf) of CpGV-M (KM217575) as a reference.

| **ID** | **Start** | **End** | **Strand** | **Length** | **M1** | **M2** | **M3** | **S1** | **S2** | **S3** | **E1** | **E2** | **E3** | **K2** |
| --- | --- | --- | --- | --- | --- | --- | --- | --- | --- | --- | --- | --- | --- | --- |
| **orf1** | 1 | 747 | + | 747 | 16 | 9 | 57 | 34 | 19 | 144 | 231 | 163 | 490 | 50 |
| **orf2** | 749 | 1273 | - | 525 | 6 | 14 | 39 | 14 | 46 | 25 | 140 | 150 | 269 | 105 |
| **orf3** | 1254 | 2093 | + | 840 | 75 | 62 | 406 | 207 | 237 | 596 | 1497 | 1030 | 2454 | 468 |
| **orf4** | 2173 | 2739 | - | 567 | 67 | 23 | 288 | 74 | 140 | 184 | 1105 | 890 | 1621 | 372 |
| **orf5** | 2729 | 2971 | + | 243 | 11 | 14 | 38 | 28 | 40 | 51 | 256 | 214 | 444 | 39 |
| **orf6** | 3122 | 3298 | + | 177 | 13 | 16 | 27 | 14 | 24 | 30 | 167 | 129 | 332 | 42 |
| **orf7** | 3391 | 4857 | - | 1467 | 25 | 25 | 96 | 53 | 51 | 127 | 523 | 497 | 922 | 58 |
| **orf8** | 4963 | 5541 | + | 579 | 143 | 79 | 541 | 202 | 417 | 503 | 2600 | 1724 | 3325 | 493 |
| **orf9** | 5581 | 5886 | - | 306 | 0 | 0 | 9 | 7 | 3 | 17 | 66 | 11 | 61 | 10 |
| **orf10** | 6027 | 7811 | - | 1785 | 78 | 61 | 422 | 120 | 261 | 543 | 2208 | 1371 | 3056 | 366 |
| **orf11** | 7934 | 8935 | + | 1002 | 28 | 37 | 114 | 63 | 91 | 294 | 501 | 338 | 735 | 158 |
| **orf12** | 9015 | 9248 | + | 234 | 23 | 12 | 101 | 37 | 68 | 107 | 240 | 155 | 397 | 175 |
| **orf13** | 9318 | 10073 | - | 756 | 16 | 3 | 97 | 24 | 34 | 101 | 341 | 252 | 600 | 67 |
| **orf14** | 10145 | 10399 | - | 255 | 5 | 4 | 29 | 10 | 13 | 45 | 95 | 99 | 185 | 48 |
| **orf15** | 10400 | 11773 | - | 1374 | 7 | 13 | 92 | 33 | 30 | 62 | 221 | 176 | 344 | 62 |
| **orf16** | 12145 | 12735 | - | 591 | 12 | 11 | 66 | 31 | 48 | 105 | 310 | 264 | 470 | 135 |
| **orf17** | 12865 | 13692 | + | 828 | 142 | 82 | 746 | 180 | 439 | 573 | 2328 | 1696 | 3390 | 1259 |
| **orf18** | 13730 | 14797 | - | 1068 | 7 | 7 | 42 | 16 | 12 | 56 | 151 | 146 | 308 | 51 |
| **orf19** | 15171 | 15398 | + | 228 | 45 | 9 | 67 | 25 | 65 | 48 | 361 | 266 | 493 | 138 |
| **orf20** | 15458 | 16165 | - | 708 | 2 | 3 | 11 | 9 | 3 | 34 | 53 | 40 | 123 | 10 |
| **orf21** | 16434 | 16613 | - | 180 | 5 | 0 | 53 | 15 | 17 | 34 | 86 | 48 | 119 | 84 |
| **orf22** | 16836 | 17879 | + | 1044 | 4 | 4 | 19 | 11 | 2 | 42 | 71 | 122 | 268 | 22 |
| **orf23** | 17970 | 18428 | + | 459 | 5 | 8 | 34 | 32 | 15 | 60 | 142 | 173 | 347 | 17 |
| **orf24** | 18571 | 19719 | - | 1149 | 113 | 79 | 542 | 138 | 286 | 417 | 1701 | 1173 | 2148 | 505 |
| **orf25** | 20165 | 20329 | - | 165 | 2 | 5 | 48 | 0 | 4 | 2 | 0 | 2 | 2 | 3 |
| **orf26** | 20328 | 21314 | + | 987 | 4 | 3 | 29 | 18 | 6 | 28 | 45 | 59 | 116 | 8 |
| **orf27** | 20358 | 21827 | - | 1470 | 70 | 36 | 234 | 80 | 135 | 215 | 535 | 407 | 760 | 352 |
| **orf28/29** | 22688 | 24058 | + | 1371 | 39 | 27 | 140 | 71 | 97 | 129 | 580 | 519 | 958 | 187 |
| **orf30** | 24629 | 25174 | + | 546 | 52 | 91 | 520 | 98 | 226 | 391 | 1338 | 1199 | 2537 | 779 |
| **orf31** | 25306 | 27099 | + | 1794 | 25 | 69 | 175 | 55 | 102 | 206 | 881 | 880 | 2265 | 364 |
| **orf32** | 27325 | 28665 | + | 1341 | 224 | 303 | 1693 | 528 | 813 | 1617 | 5384 | 4627 | 10691 | 3517 |
| **orf33** | 28737 | 29621 | - | 885 | 14 | 14 | 74 | 22 | 29 | 46 | 300 | 199 | 392 | 71 |
| **orf34** | 29664 | 30353 | - | 690 | 3 | 2 | 24 | 0 | 5 | 9 | 63 | 65 | 91 | 36 |
| **orf35** | 30247 | 30822 | + | 576 | 0 | 2 | 5 | 5 | 4 | 18 | 25 | 20 | 47 | 2 |
| **orf36b** | 30901 | 31083 | - | 183 | 0 | 1 | 0 | 0 | 1 | 2 | 5 | 9 | 11 | 5 |
| **orf36a** | 30998 | 31162 | - | 165 | 0 | 0 | 0 | 0 | 0 | 0 | 5 | 5 | 3 | 0 |
| **orf37** | 31205 | 33439 | - | 2235 | 2 | 0 | 10 | 2 | 3 | 15 | 44 | 43 | 81 | 35 |
| **orf39** | 33481 | 33798 | + | 318 | 5 | 3 | 38 | 6 | 14 | 18 | 151 | 61 | 135 | 27 |
| **orf40** | 33851 | 34180 | - | 330 | 88 | 29 | 193 | 50 | 77 | 198 | 753 | 719 | 1257 | 400 |
| **orf41** | 34321 | 34836 | + | 516 | 32 | 18 | 122 | 70 | 73 | 153 | 642 | 448 | 789 | 263 |
| **orf42** | 34928 | 35176 | + | 249 | 7 | 7 | 33 | 16 | 18 | 27 | 87 | 97 | 138 | 128 |
| **orf43** | 35223 | 35567 | - | 345 | 38 | 11 | 59 | 35 | 64 | 98 | 353 | 369 | 695 | 208 |
| **orf44** | 35632 | 36255 | - | 624 | 369 | 439 | 2589 | 823 | 1823 | 1725 | 9698 | 5940 | 13905 | 4731 |
| **orf45** | 36314 | 36778 | - | 465 | 153 | 264 | 949 | 317 | 728 | 591 | 3771 | 2992 | 6156 | 1973 |
| **orf46** | 36836 | 38473 | - | 1638 | 42 | 98 | 290 | 105 | 219 | 206 | 1761 | 1584 | 3322 | 419 |
| **orf47** | 38480 | 39289 | + | 810 | 3 | 8 | 38 | 8 | 15 | 26 | 126 | 80 | 155 | 27 |
| **orf48** | 39333 | 40451 | + | 1119 | 13 | 7 | 53 | 13 | 25 | 35 | 314 | 238 | 406 | 49 |
| **orf49** | 40448 | 40837 | - | 390 | 5 | 0 | 17 | 7 | 16 | 19 | 49 | 39 | 74 | 23 |
| **orf50/51** | 40773 | 44090 | + | 3318 | 122 | 111 | 665 | 284 | 343 | 644 | 3699 | 2358 | 4551 | 612 |
| **orf52b** | 43709 | 44071 | - | 363 | 4 | 3 | 2 | 1 | 5 | 7 | 28 | 13 | 34 | 11 |
| **orf52a** | 44099 | 44839 | - | 741 | 4 | 8 | 32 | 5 | 17 | 32 | 136 | 101 | 231 | 27 |
| **orf53** | 44849 | 44995 | + | 147 | 1 | 0 | 15 | 5 | 8 | 11 | 59 | 45 | 85 | 15 |
| **orf54** | 45071 | 45355 | - | 285 | 117 | 69 | 629 | 160 | 340 | 570 | 2030 | 1509 | 3657 | 912 |
| **orf55** | 45434 | 46498 | + | 1065 | 31 | 24 | 151 | 48 | 79 | 156 | 823 | 680 | 1075 | 184 |
| **orf56** | 46505 | 46714 | + | 210 | 9 | 10 | 49 | 18 | 28 | 39 | 246 | 139 | 270 | 53 |
| **orf57** | 46791 | 47516 | - | 726 | 839 | 1065 | 4149 | 1585 | 3382 | 3154 | 22359 | 17341 | 31970 | 3215 |
| **orf58** | 47467 | 47871 | - | 405 | 90 | 150 | 434 | 92 | 231 | 157 | 2251 | 1922 | 3511 | 265 |
| **orf59** | 47826 | 48224 | - | 399 | 2 | 7 | 14 | 1 | 13 | 10 | 115 | 95 | 227 | 20 |
| **orf60** | 48598 | 50664 | - | 2067 | 12 | 15 | 121 | 33 | 54 | 170 | 411 | 305 | 796 | 81 |
| **orf61** | 50883 | 51095 | - | 213 | 7 | 3 | 29 | 18 | 22 | 35 | 145 | 95 | 186 | 26 |
| **orf62** | 51067 | 51636 | - | 570 | 25 | 4 | 231 | 218 | 77 | 227 | 366 | 262 | 724 | 232 |
| **orf63** | 51774 | 51941 | + | 168 | 10 | 5 | 38 | 9 | 6 | 17 | 67 | 68 | 75 | 39 |
| **orf64** | 52654 | 53346 | + | 693 | 163 | 55 | 631 | 266 | 264 | 925 | 1761 | 1280 | 2772 | 660 |
| **orf65** | 53444 | 53683 | - | 240 | 0 | 0 | 11 | 4 | 6 | 22 | 49 | 14 | 61 | 14 |
| **orf66** | 53838 | 54059 | + | 222 | 73 | 31 | 339 | 70 | 113 | 511 | 828 | 883 | 1838 | 500 |
| **orf67** | 54134 | 54397 | - | 264 | 30 | 18 | 99 | 15 | 54 | 37 | 340 | 254 | 564 | 97 |
| **orf68** | 54369 | 55751 | + | 1383 | 38 | 51 | 178 | 63 | 80 | 278 | 942 | 757 | 1515 | 147 |
| **orf69** | 55790 | 56452 | + | 663 | 90 | 69 | 251 | 131 | 307 | 374 | 2059 | 1225 | 2540 | 215 |
| **orf70** | 56526 | 57080 | - | 555 | 3 | 5 | 11 | 1 | 2 | 2 | 31 | 37 | 115 | 22 |
| **orf71** | 57150 | 57761 | + | 612 | 9 | 4 | 63 | 24 | 21 | 92 | 522 | 241 | 618 | 47 |
| **orf72** | 57800 | 58144 | - | 345 | 199 | 164 | 960 | 132 | 418 | 391 | 4591 | 2890 | 6383 | 863 |
| **orf73** | 58338 | 58934 | - | 597 | 147 | 216 | 942 | 271 | 534 | 680 | 4435 | 3428 | 8332 | 1007 |
| **orf74** | 58915 | 59622 | - | 708 | 25 | 51 | 178 | 36 | 92 | 166 | 816 | 533 | 1599 | 258 |
| **orf75** | 59748 | 61364 | + | 1617 | 11 | 17 | 63 | 38 | 21 | 148 | 446 | 312 | 763 | 78 |
| **orf76** | 61523 | 62203 | - | 681 | 31 | 32 | 332 | 91 | 108 | 201 | 1344 | 1126 | 2003 | 370 |
| **orf77** | 62271 | 62582 | - | 312 | 94 | 49 | 345 | 44 | 190 | 164 | 1565 | 1063 | 2134 | 420 |
| **orf78** | 62591 | 62818 | - | 228 | 11 | 6 | 48 | 10 | 28 | 29 | 151 | 105 | 300 | 95 |
| **orf79** | 62842 | 63312 | + | 471 | 2 | 8 | 25 | 9 | 16 | 40 | 133 | 83 | 250 | 46 |
| **orf80** | 63309 | 63614 | - | 306 | 31 | 52 | 159 | 49 | 131 | 85 | 683 | 566 | 1254 | 385 |
| **orf81** | 63693 | 64565 | - | 873 | 319 | 261 | 1936 | 571 | 1091 | 1361 | 6883 | 5017 | 10127 | 2151 |
| **orf82b** | 64592 | 64849 | - | 258 | 22 | 12 | 103 | 31 | 83 | 73 | 551 | 387 | 745 | 123 |
| **orf82a** | 64773 | 65465 | - | 693 | 34 | 36 | 156 | 48 | 109 | 157 | 692 | 619 | 1245 | 176 |
| **orf83** | 65355 | 66674 | + | 1320 | 8 | 19 | 80 | 22 | 37 | 109 | 270 | 238 | 528 | 56 |
| **orf84** | 66706 | 67035 | + | 330 | 14 | 31 | 63 | 26 | 49 | 61 | 302 | 251 | 552 | 118 |
| **orf85** | 67094 | 68236 | + | 1143 | 154 | 279 | 1141 | 352 | 718 | 911 | 3649 | 3009 | 6585 | 2014 |
| **orf86** | 68268 | 68417 | + | 150 | 266 | 333 | 2587 | 545 | 1356 | 1322 | 6602 | 4996 | 12219 | 4009 |
| **orf87** | 68518 | 69246 | - | 729 | 36 | 17 | 133 | 34 | 118 | 73 | 692 | 505 | 913 | 182 |
| **orf88** | 69070 | 70101 | + | 1032 | 19 | 24 | 182 | 68 | 108 | 171 | 665 | 572 | 1243 | 257 |
| **orf89** | 70269 | 70754 | - | 486 | 0 | 2 | 12 | 1 | 17 | 9 | 76 | 72 | 124 | 23 |
| **orf90** | 70738 | 74133 | + | 3396 | 239 | 242 | 1199 | 321 | 664 | 892 | 5088 | 3749 | 8479 | 1455 |
| **orf91** | 74245 | 74886 | - | 642 | 1 | 0 | 20 | 9 | 7 | 51 | 150 | 102 | 296 | 24 |
| **orf92** | 74976 | 75461 | - | 486 | 0 | 0 | 5 | 2 | 0 | 4 | 40 | 22 | 48 | 11 |
| **orf93** | 75525 | 76280 | + | 756 | 4 | 4 | 21 | 4 | 11 | 22 | 83 | 88 | 185 | 16 |
| **orf94** | 76324 | 77055 | - | 732 | 110 | 90 | 620 | 143 | 359 | 341 | 3022 | 1819 | 3320 | 387 |
| **orf95** | 77060 | 78502 | - | 1443 | 39 | 48 | 169 | 26 | 138 | 123 | 1057 | 668 | 1194 | 153 |
| **orf96** | 78574 | 79431 | + | 858 | 168 | 233 | 1146 | 205 | 524 | 674 | 4486 | 3709 | 7495 | 1851 |
| **orf97** | 79573 | 80439 | + | 867 | 35 | 61 | 133 | 83 | 191 | 200 | 925 | 860 | 1736 | 378 |
| **orf98** | 80753 | 81238 | + | 486 | 114 | 101 | 394 | 241 | 389 | 463 | 2188 | 1648 | 2852 | 584 |
| **orf99** | 81329 | 82513 | - | 1185 | 30 | 25 | 163 | 46 | 90 | 121 | 685 | 480 | 890 | 134 |
| **orf100** | 82545 | 82871 | + | 327 | 1 | 0 | 11 | 2 | 4 | 17 | 51 | 50 | 89 | 15 |
| **orf101** | 82906 | 84903 | - | 1998 | 1 | 13 | 20 | 7 | 24 | 25 | 111 | 107 | 123 | 26 |
| **orf102** | 84884 | 85534 | + | 651 | 28 | 14 | 64 | 29 | 43 | 119 | 458 | 348 | 635 | 128 |
| **orf103** | 85509 | 86084 | + | 576 | 13 | 14 | 65 | 34 | 49 | 96 | 330 | 261 | 562 | 142 |
| **orf104** | 86110 | 86979 | + | 870 | 13 | 9 | 28 | 8 | 8 | 51 | 146 | 123 | 315 | 49 |
| **orf105** | 87059 | 87319 | + | 261 | 5 | 2 | 16 | 2 | 5 | 23 | 77 | 37 | 103 | 25 |
| **orf106** | 87276 | 88412 | + | 1137 | 94 | 92 | 381 | 119 | 272 | 333 | 1924 | 1461 | 2780 | 601 |
| **orf107** | 88508 | 88762 | + | 255 | 1 | 0 | 7 | 4 | 2 | 10 | 40 | 30 | 86 | 9 |
| **orf108** | 88874 | 89320 | + | 447 | 11 | 5 | 61 | 27 | 13 | 79 | 239 | 170 | 478 | 43 |
| **orf109** | 89415 | 89990 | - | 576 | 174 | 93 | 984 | 285 | 368 | 727 | 1827 | 1633 | 3716 | 1571 |
| **orf110** | 90352 | 90729 | - | 378 | 53 | 32 | 184 | 99 | 115 | 187 | 1052 | 709 | 1657 | 298 |
| **orf111** | 90849 | 94004 | - | 3156 | 115 | 79 | 309 | 115 | 250 | 275 | 1902 | 1591 | 3549 | 527 |
| **orf112** | 93946 | 96102 | + | 2157 | 62 | 54 | 389 | 112 | 175 | 269 | 1509 | 1218 | 2615 | 329 |
| **orf113** | 96313 | 97374 | - | 1062 | 121 | 124 | 680 | 227 | 473 | 496 | 3448 | 2445 | 4701 | 557 |
| **orf114** | 97343 | 97723 | + | 381 | 10 | 11 | 49 | 22 | 28 | 75 | 142 | 129 | 334 | 58 |
| **orf115** | 97845 | 98351 | + | 507 | 146 | 73 | 215 | 105 | 151 | 252 | 1769 | 1237 | 1825 | 134 |
| **orf116** | 98533 | 99360 | + | 828 | 4 | 7 | 27 | 11 | 20 | 31 | 172 | 153 | 339 | 42 |
| **orf117** | 99335 | 100834 | + | 1500 | 43 | 66 | 251 | 70 | 150 | 172 | 1327 | 1086 | 2354 | 351 |
| **orf118** | 100870 | 101355 | + | 486 | 50 | 61 | 298 | 61 | 148 | 298 | 1276 | 983 | 2330 | 362 |
| **orf119** | 101468 | 101956 | + | 489 | 82 | 60 | 526 | 149 | 255 | 465 | 1799 | 1263 | 3382 | 539 |
| **orf120** | 102007 | 103719 | - | 1713 | 54 | 77 | 399 | 72 | 161 | 227 | 1042 | 888 | 2008 | 596 |
| **orf121** | 104019 | 104234 | + | 216 | 24 | 13 | 73 | 22 | 40 | 84 | 403 | 351 | 707 | 57 |
| **orf122** | 104328 | 104528 | + | 201 | 23 | 19 | 165 | 70 | 101 | 119 | 553 | 476 | 946 | 155 |
| **orf123** | 104618 | 105820 | - | 1203 | 105 | 386 | 1622 | 464 | 636 | 1113 | 5803 | 4999 | 13010 | 2061 |
| **orf124** | 105973 | 106272 | + | 300 | 87 | 99 | 734 | 269 | 376 | 499 | 1770 | 1766 | 2834 | 1842 |
| **orf125** | 106435 | 107631 | + | 1197 | 36 | 63 | 117 | 62 | 143 | 130 | 956 | 747 | 1687 | 131 |
| **orf126** | 107555 | 108928 | + | 1374 | 166 | 148 | 649 | 283 | 636 | 560 | 3530 | 2130 | 4951 | 602 |
| **orf127** | 109017 | 110891 | - | 1875 | 28 | 34 | 185 | 69 | 100 | 159 | 885 | 681 | 1411 | 230 |
| **orf128** | 111019 | 112107 | + | 1089 | 74 | 62 | 316 | 124 | 199 | 346 | 1176 | 822 | 1934 | 455 |
| **orf129/130** | 112140 | 113120 | - | 981 | 125 | 133 | 837 | 233 | 573 | 611 | 3057 | 2477 | 5107 | 1020 |
| **orf131** | 113198 | 115819 | - | 2622 | 58 | 96 | 338 | 118 | 253 | 247 | 1472 | 1326 | 2798 | 530 |
| **orf132** | 115907 | 116302 | + | 396 | 220 | 190 | 1389 | 440 | 815 | 804 | 3237 | 2562 | 4786 | 1389 |
| **orf133** | 116364 | 116552 | - | 189 | 11 | 10 | 81 | 23 | 35 | 83 | 365 | 236 | 518 | 154 |
| **orf134** | 116542 | 116943 | + | 402 | 11 | 15 | 99 | 43 | 51 | 58 | 303 | 194 | 457 | 139 |
| **orf135** | 117012 | 118142 | - | 1131 | 183 | 312 | 1385 | 477 | 1155 | 980 | 6020 | 3872 | 8833 | 1765 |
| **orf136** | 118148 | 118369 | - | 222 | 22 | 18 | 104 | 27 | 92 | 110 | 412 | 332 | 716 | 140 |
| **orf137** | 118320 | 118589 | + | 270 | 0 | 8 | 34 | 17 | 16 | 19 | 164 | 142 | 382 | 61 |
| **orf138** | 118447 | 119445 | + | 999 | 41 | 62 | 349 | 93 | 220 | 225 | 1577 | 1159 | 2921 | 429 |
| **orf139** | 119335 | 119655 | - | 321 | 0 | 5 | 1 | 0 | 6 | 8 | 36 | 37 | 93 | 15 |
| **orf140** | 119756 | 120799 | + | 1044 | 229 | 341 | 2410 | 600 | 1065 | 1395 | 9196 | 6279 | 15628 | 2802 |
| **orf141** | 120882 | 122336 | - | 1455 | 108 | 162 | 670 | 232 | 426 | 498 | 5159 | 3072 | 5423 | 493 |
| **orf142** | 122354 | 122557 | + | 204 | 2 | 3 | 11 | 9 | 20 | 44 | 60 | 57 | 144 | 24 |
| **orf143** | 122530 | 123441 | + | 912 | 53 | 24 | 166 | 106 | 164 | 369 | 730 | 555 | 1212 | 382 |
