# Supplementary material for: Transcriptome analysis of CpGV in midguts of type II resistant codling moth larvae and identification of contaminant infections by SNP mapping of RNA-Seq data
Source: J Virol. 2024 Jun 27;98(7):e00537-24. doi: 10.1128/jvi.00537-24 (PMC11265400; doi:10.1128/jvi.00537-24)
Supplement: Table S3 — TPM value of every gene. [file jvi.00537-24-s0005.docx]

**TABLE S3** Calculated TPM values of each gene from different groups of RNA-Seq samples from infections with CpGV-M (M1-M3), CpGV-S (S1, S2S3), and CpGV-E2 (E1-E3). Given are the open reading frame number (ID) and the gene name, promoter motif(s) and putative function (as far as known) (see Wennmann et al., 2021. Journal of General Virology 102(3), 001566. DOI 10.1099/jgv.0.001566. *Abbreviations: e=early, l=late, struc = structural protein, reg = regulatory protein, aux = auxiliary protein.

| **ID** | **Gene name** | **Promoter*** | **Function*** | **M1-M3** | **S1** | **S2S3** | **E1-E3** |
| --- | --- | --- | --- | --- | --- | --- | --- |
| **orf1** | *granulin* | l | struc | 16.36 | 15.74 | 41.45 | 116.26 |
| **orf2** |  | e, l |  | 16.75 | 9.22 | 25.69 | 104.60 |
| **orf3** | *pk1* | e | struc | 96.36 | 85.21 | 188.37 | 582.56 |
| **orf4** |  | e, l |  | 99.38 | 45.13 | 108.55 | 626.53 |
| **orf5** |  |  |  | 38.65 | 39.84 | 71.14 | 369.52 |
| **orf6** |  | l |  | 47.16 | 27.35 | 57.95 | 348.57 |
| **orf7** | *ie-1* | e | reg | 14.84 | 12.49 | 23.05 | 130.05 |
| **orf8** | *ac146* | e | struc | 196.43 | 120.63 | 301.83 | 1297.85 |
| **orf9** | *ac145* | e, l | struc | 4.38 | 7.91 | 12.42 | 44.31 |
| **orf10** | *chitinase* | e | aux | 46.85 | 23.24 | 85.56 | 365.18 |
| **orf11** | *cathepsin* | l | aux | 26.63 | 21.74 | 72.99 | 154.33 |
| **orf12** |  | l |  | 86.64 | 54.67 | 142.06 | 332.51 |
| **orf13** | *gp37* | l | aux | 22.87 | 10.98 | 33.92 | 155.03 |
| **orf14** | *odv-e18* | l | struc | 22.21 | 13.56 | 43.21 | 146.02 |
| **orf15** | *p49* | e, l | struc | 12.15 | 8.30 | 12.72 | 52.98 |
| **orf16** |  | e |  | 22.45 | 18.14 | 49.18 | 173.55 |
| **orf17** | *iap-3* | e | reg | 174.63 | 75.17 | 232.17 | 879.67 |
| **orf18** | *odv-e56* | e, l | struc | 7.82 | 5.18 | 12.09 | 55.65 |
| **orf19** | *orf15R* | e |  | 79.11 | 37.91 | 94.15 | 482.60 |
| **orf20** | *orf16L* | l |  | 3.37 | 4.40 | 9.93 | 29.97 |
| **orf21** | *orf17L* |  |  | 48.03 | 28.81 | 53.82 | 138.09 |
| **orf22** | *orf17R* | l | struc | 3.86 | 3.64 | 8.01 | 43.38 |
| **orf23** | *pe/pp34* | e, l | struc | 15.26 | 24.11 | 31.04 | 141.69 |
| **orf24** | *pe38* | e | reg | 95.22 | 41.53 | 116.22 | 429.39 |
| **orf25** |  | e |  | 49.69 | 0.00 | 6.91 | 2.38 |
| **orf26** |  | l |  | 5.44 | 6.31 | 6.54 | 21.90 |
| **orf27** |  | e |  | 34.48 | 18.82 | 45.23 | 113.75 |
| **orf28/29** | *1* | e |  | 22.40 | 17.91 | 31.31 | 147.40 |
| **orf30** |  | e |  | 181.01 | 62.06 | 214.66 | 912.97 |
| **orf31** | *f-protein* | e | struc | 22.35 | 10.60 | 32.61 | 220.47 |
| **orf32** |  | l |  | 246.77 | 136.14 | 344.22 | 1516.64 |
| **orf33** |  | l |  | 17.18 | 8.60 | 16.10 | 98.91 |
| **orf34** |  | e |  | 6.26 | 0.00 | 3.85 | 31.18 |
| **orf35** | *pif-3* | e, l | struc | 1.81 | 3.00 | 7.26 | 15.69 |
| **orf36b** |  | e |  | 0.81 | 0.00 | 3.11 | 13.42 |
| **orf36a** |  | e |  | 0.00 | 0.00 | 0.00 | 7.74 |
| **orf37** | *odv-e66* | l | struc | 0.80 | 0.31 | 1.53 | 7.38 |
| **orf39** |  | l |  | 21.56 | 6.52 | 19.12 | 107.20 |
| **orf40** |  |  |  | 140.03 | 52.39 | 158.30 | 812.44 |
| **orf41** | *lef-2* | e | reg | 49.69 | 46.91 | 83.20 | 357.75 |
| **orf42** | *orf35a* | e |  | 28.14 | 22.22 | 34.33 | 127.04 |
| **orf43** |  | e, l |  | 46.66 | 35.08 | 89.20 | 403.51 |
| **orf44** | *orf36L* | e, l |  | 811.49 | 456.04 | 1080.08 | 4651.25 |
| **orf45** |  | e |  | 437.89 | 235.72 | 538.83 | 2729.45 |
| **orf46** | *mp-nase* |  | struc | 39.13 | 22.16 | 49.29 | 399.87 |
| **orf47** | *p13* | l | struc | 9.02 | 3.41 | 9.62 | 43.78 |
| **orf48** | *pif-1* |  | struc | 9.72 | 4.02 | 10.19 | 84.11 |
| **orf49** |  | l |  | 8.41 | 6.21 | 17.05 | 40.81 |
| **orf50/51** |  | l |  | 40.34 | 29.60 | 56.51 | 314.09 |
| **orf52b** | *ac106/107* | l |  | 3.70 | 0.95 | 6.28 | 20.30 |
| **orf52a** |  | e |  | 8.85 | 2.33 | 12.56 | 62.05 |
| **orf53** | *ac110* | e |  | 16.22 | 11.76 | 24.55 | 126.31 |
| **orf54** | *ubiquitin* |  | aux | 426.27 | 194.12 | 606.53 | 2480.54 |
| **orf55** | *odv-ec43* | e, l | struc | 28.83 | 15.58 | 41.92 | 237.81 |
| **orf56** | *ac108* | l | struc | 48.27 | 29.64 | 60.61 | 306.42 |
| **orf57** | *pp31/39K* | e | reg | 1242.81 | 754.88 | 1710.14 | 9698.41 |
| **orf58** | *lef-11* |  | reg | 248.07 | 78.54 | 181.98 | 1863.94 |
| **orf59** | *sod* | l | aux | 8.59 | 0.87 | 10.95 | 107.60 |
| **orf60** | *p74* | l | struc | 10.67 | 5.52 | 20.59 | 71.86 |
| **orf61** |  | l |  | 27.29 | 29.22 | 50.83 | 196.49 |
| **orf62** |  | e, l |  | 67.99 | 132.24 | 101.31 | 233.02 |
| **orf63** | *bro* | e | reg | 47.03 | 18.52 | 26.01 | 122.80 |
| **orf64** |  | e, l |  | 182.62 | 132.72 | 325.92 | 824.08 |
| **orf65** |  | l |  | 6.83 | 5.76 | 22.16 | 50.76 |
| **orf66** | *ptp-2* |  | struc | 297.46 | 109.03 | 533.94 | 1570.55 |
| **orf67** |  | l |  | 83.00 | 19.65 | 65.48 | 430.93 |
| **orf68** | *p47/pif-5* | e | reg | 28.78 | 15.75 | 49.17 | 228.31 |
| **orf69** |  | l |  | 92.18 | 68.32 | 195.12 | 862.99 |
| **orf70** |  | l |  | 5.10 | 0.62 | 1.37 | 32.39 |
| **orf71** | *p24capsid* | l | struc | 18.51 | 13.56 | 35.07 | 221.69 |
| **orf72** |  |  |  | 571.63 | 132.29 | 445.44 | 3947.93 |
| **orf73** | *38.7K* | e |  | 325.84 | 156.96 | 386.28 | 2665.06 |
| **orf74** | *lef-1* | e | reg | 53.48 | 17.58 | 69.22 | 409.07 |
| **orf75** |  |  |  | 8.39 | 8.13 | 19.85 | 92.41 |
| **orf76** | *fgf-1* | e | aux | 86.46 | 46.20 | 86.19 | 645.28 |
| **orf77** |  | e |  | 233.15 | 48.76 | 215.53 | 1499.46 |
| **orf78** |  | e |  | 42.50 | 15.17 | 47.49 | 239.57 |
| **orf79** |  | l |  | 11.08 | 6.61 | 22.59 | 97.20 |
| **orf80** | *lef-6* | e | reg | 117.89 | 55.37 | 134.09 | 803.60 |
| **orf81** | *dbp* | e | reg | 429.60 | 226.15 | 533.54 | 2478.80 |
| **orf82b** | *82a* | e |  | 79.15 | 41.55 | 114.86 | 640.86 |
| **orf82a** | *82b* | e |  | 48.61 | 23.95 | 72.91 | 362.35 |
| **orf83** | *p45(p48)* | e | struc | 12.08 | 5.76 | 21.01 | 77.11 |
| **orf84** | *p12* | l | struc | 48.78 | 27.24 | 63.32 | 328.96 |
| **orf85** | *bv/odv-c42 (p40)* | e, l | struc | 205.27 | 106.48 | 270.73 | 1138.26 |
| **orf86** | *p6.9* | l | struc | 3166.11 | 1256.29 | 3391.38 | 15598.96 |
| **orf87** | *lef-5* |  | reg | 38.03 | 16.13 | 49.77 | 284.35 |
| **orf88** | *38k* |  | struc | 32.50 | 22.78 | 51.35 | 236.09 |
| **orf89** | *pif-4* | l | struc | 4.29 | 0.71 | 10.16 | 54.98 |
| **orf90** | *helicase* | l | reg | 73.74 | 32.68 | 87.04 | 500.93 |
| **orf91** | *odv-e25* | e, l | struc | 4.88 | 4.85 | 17.16 | 83.86 |
| **orf92** | *p18* | l | struc | 1.53 | 1.42 | 1.56 | 22.24 |
| **orf93** | *p33* | e, l | struc | 5.72 | 1.83 | 8.29 | 46.26 |
| **orf94** | *iap* | l | reg | 166.98 | 67.55 | 181.65 | 1095.30 |
| **orf95** | *lef-4* | l | reg | 26.45 | 6.23 | 34.36 | 198.73 |
| **orf96** | *vp39* | l | struc | 268.77 | 82.61 | 265.23 | 1796.53 |
| **orf97** | *odv-ec27* | e, l | struc | 39.37 | 33.10 | 85.67 | 398.98 |
| **orf98** | *ptp* | e | struc | 186.79 | 171.46 | 333.01 | 1351.95 |
| **orf99** |  | e |  | 27.42 | 13.42 | 33.82 | 170.37 |
| **orf100** |  | l |  | 5.47 | 2.11 | 12.20 | 57.08 |
| **orf101** | *vp91* | e, l | struc | 2.54 | 1.21 | 4.66 | 16.77 |
| **orf102** | *tlp20* | l |  | 24.27 | 15.40 | 47.27 | 217.46 |
| **orf103** | *ac81* | l |  | 23.81 | 20.41 | 47.82 | 196.66 |
| **orf104** | *gp41* | l | struc | 8.57 | 3.18 | 12.88 | 65.95 |
| **orf105** | *ac78* | l |  | 13.14 | 2.65 | 20.38 | 81.68 |
| **orf106** |  | e, l |  | 74.34 | 36.19 | 101.08 | 532.69 |
| **orf107** |  |  |  | 4.68 | 5.42 | 8.94 | 60.10 |
| **orf108** | *ac75* | l | struc | 25.68 | 20.89 | 39.10 | 194.95 |
| **orf109** |  |  |  | 323.75 | 171.08 | 361.12 | 1223.94 |
| **orf110** |  | e |  | 106.08 | 90.56 | 151.77 | 888.34 |
| **orf111** | *dnapol* |  | reg | 23.76 | 12.60 | 31.60 | 219.21 |
| **orf112** | *desmoplakin* | e | struc | 34.90 | 17.95 | 39.10 | 243.31 |
| **orf113** | *lef-3* | e | reg | 129.83 | 73.91 | 173.32 | 980.02 |
| **orf114** | *pif-6* | e | struc | 27.39 | 19.97 | 51.35 | 156.00 |
| **orf115** |  | e |  | 127.60 | 71.61 | 150.99 | 936.11 |
| **orf116** | *iap-5* | e | aux | 6.84 | 4.59 | 11.70 | 78.78 |
| **orf117** | *lef-9* | l | reg | 35.78 | 16.14 | 40.78 | 312.21 |
| **orf118** | *fp25k* | l | struc | 125.45 | 43.40 | 174.32 | 927.64 |
| **orf119** |  | e |  | 203.63 | 105.36 | 279.69 | 1294.63 |
| **orf120** | *DNA ligase* |  | reg | 46.12 | 14.53 | 43.03 | 225.85 |
| **orf121** |  |  |  | 75.91 | 35.22 | 109.05 | 664.50 |
| **orf122** |  | e |  | 153.51 | 120.42 | 207.91 | 965.32 |
| **orf123** | *fgf* |  | aux | 261.82 | 133.36 | 276.17 | 1944.60 |
| **orf124** |  | e |  | 457.13 | 310.04 | 554.04 | 2086.02 |
| **orf125** | *alk-exo* | e | aux | 26.90 | 17.91 | 43.32 | 278.23 |
| **orf126** | *helicase-2* | e | reg | 104.47 | 71.22 | 165.35 | 758.70 |
| **orf127** | *rr1* | e | reg | 19.64 | 12.72 | 26.24 | 155.98 |
| **orf128** | *rr2a* |  | reg | 61.87 | 39.37 | 95.07 | 354.72 |
| **orf129/130** |  | e | reg | 166.39 | 82.12 | 229.27 | 1065.65 |
| **orf131** | *lef-8* | e | reg | 27.97 | 15.56 | 36.22 | 209.67 |
| **orf132** |  |  |  | 677.19 | 384.19 | 776.62 | 2626.00 |
| **orf133** |  | e |  | 80.45 | 42.08 | 118.60 | 581.66 |
| **orf134** |  | l |  | 46.35 | 36.99 | 51.51 | 233.14 |
| **orf135** |  | l |  | 247.78 | 145.83 | 358.59 | 1626.52 |
| **orf136** |  | l |  | 96.69 | 42.05 | 172.84 | 646.10 |
| **orf137** | *lef-10* | e, l | aux | 23.19 | 21.77 | 24.62 | 250.34 |
| **orf138** | *vp1054* | e | struc | 67.44 | 32.19 | 84.62 | 556.31 |
| **orf139** |  | e |  | 2.79 | 0.00 | 8.28 | 50.80 |
| **orf140** | *fgf-3* | e | aux | 425.49 | 198.72 | 447.60 | 2926.86 |
| **orf141** | *egt* | e | aux | 96.30 | 55.13 | 120.63 | 921.93 |
| **orf142** |  | e |  | 11.69 | 15.25 | 59.59 | 125.69 |
| **orf143** |  |  |  | 39.72 | 40.19 | 111.02 | 268.98 |
