# Supplementary material for: Transcriptome analysis of CpGV in midguts of type II resistant codling moth larvae and identification of contaminant infections by SNP mapping of RNA-Seq data
Source: J Virol. 2024 Jun 27;98(7):e00537-24. doi: 10.1128/jvi.00537-24 (PMC11265400; doi:10.1128/jvi.00537-24)
Supplement: Table S4 — Statistical analysis of ranking position. [file jvi.00537-24-s0006.docx]

**TABLE S4** Statistical analysis of the ranking position changes in the sample groups M1-M3, S1 and S2S3. Given are the standard deviation of the mean (STDEV) and the maximum (Max) and minimum (Min) value in the rank change. The ranks positions outside the 90% percentile (>90%) are given on the bottom line.

|  | **Sample**  **M1-M3** | **Sample**  **S1** | **Sample**  **S2S3** |
| --- | --- | --- | --- |
| **Mean** | 0.00 | 0.00 | 0.00 |
| **STDEV** | 11.65 | 13.42 | 10.42 |
| **Max** | 85.00 | 58.00 | 35.00 |
| **Min** | -23.00 | -32.00 | -33.00 |
| **>90%** | < -20,> +20 | < -22, > +22 | < -17, > +17 |
