# Supplementary material for: Transcriptome analysis of CpGV in midguts of type II resistant codling moth larvae and identification of contaminant infections by SNP mapping of RNA-Seq data
Source: J Virol. 2024 Jun 27;98(7):e00537-24. doi: 10.1128/jvi.00537-24 (PMC11265400; doi:10.1128/jvi.00537-24)
Supplement: Table S5 — Change of rank position. [file jvi.00537-24-s0007.docx]

**TABLE S5:** Change of rank positions of CpGV gene transcription of CpGV-M (M1-M3) and CpGV-S (S1, S2S3) compared with CpGV-E2 (E1-E3). Given are the open reading frame (orf) number (ID) and the gene name, promoter motif(s) and putative function (as far as known) (see Wennmann et al., 2021. Journal of General Virology 102(3), 001566. DOI 10.1099/jgv.0.001566), the TPM values as well as the respective rank and the rank change (= rank of orf[i] of TPM(E1-E3) – rank of orf[i] of TPM[X], with i = 1 to 143 and X= M1-M3, S1, S2S3) in comparison to CpGV-E2. Please note that significant rank changes have different ranges for M1-M3, S1 and S2/S3 (see Table S4). Rank changes given in orange bold color represent significantly upregulated genes, whereas blue bold colors indicate downregulation.

|  |  | | | **TPM values and rank of viral genes in different experimental groups** | | | | | | | | **Change of orf rank position compared with CpGV-E2** | | |
| --- | --- | --- | --- | --- | --- | --- | --- | --- | --- | --- | --- | --- | --- | --- |
| **ID** | **Gene name** | **Promoter** | **Function** | **TPM M1-M3** | **Rank** | **TPM**  **S1** | **Rank** | **TPM S2S3** | **Rank** | **TPM E1-E3** | **Rank** | **M1-M3** | **S1** | **S2S3** |
| **orf1** | *granulin* | l | struc | 16.36 | 104 | 15.74 | 88 | 41.45 | 86 | 116.26 | 103 | -1 | 15 | **17** |
| **orf2** |  | e, l |  | 16.75 | 103 | 9.22 | 104 | 25.69 | 102 | 104.6 | 107 | 4 | 3 | 5 |
| **orf3** | *pk1* | e | struc | 96.36 | 39 | 85.21 | 25 | 188.37 | 29 | 582.56 | 44 | 5 | 19 | 15 |
| **orf4** |  | e, l |  | 99.38 | 37 | 45.13 | 43 | 108.55 | 47 | 626.53 | 43 | 6 | 0 | -4 |
| **orf5** |  |  |  | 38.65 | 73 | 39.84 | 50 | 71.14 | 61 | 369.52 | 56 | -17 | 6 | -5 |
| **orf6** |  | l |  | 47.16 | 62 | 27.35 | 64 | 57.95 | 67 | 348.57 | 61 | -1 | -3 | -6 |
| **orf7** | *ie-1* | e | reg | 14.84 | 107 | 12.49 | 100 | 23.05 | 105 | 130.05 | 98 | -9 | -2 | -7 |
| **orf8** | *ac146* | e | struc | 196.43 | 22 | 120.63 | 19 | 301.83 | 18 | 1297.85 | 20 | -2 | 1 | 2 |
| **orf9** | *ac145* | e, l | struc | 4.38 | 131 | 7.91 | 108 | 12.42 | 119 | 44.31 | 127 | -4 | 19 | 8 |
| **orf10** | *chitinase* | e | aux | 46.85 | 64 | 23.24 | 68 | 85.56 | 56 | 365.18 | 57 | -7 | -11 | 1 |
| **orf11** | *cathepsin* | l | aux | 26.63 | 87 | 21.74 | 73 | 72.99 | 59 | 154.33 | 93 | 6 | 20 | **34** |
| **orf12** |  | l |  | 86.64 | 43 | 54.67 | 38 | 142.06 | 39 | 332.51 | 62 | **19** | **24** | **23** |
| **orf13** | *gp37* | l | aux | 22.87 | 94 | 10.98 | 102 | 33.92 | 94 | 155.03 | 92 | -2 | -10 | -2 |
| **orf14** | *odv-e18* | l | struc | 22.21 | 98 | 13.56 | 95 | 43.21 | 83 | 146.02 | 95 | -3 | 0 | 12 |
| **orf15** | *p49* | e, l | struc | 12.15 | 109 | 8.3 | 106 | 12.72 | 117 | 52.98 | 123 | 14 | 17 | 6 |
| **orf16** |  | e |  | 22.45 | 95 | 18.14 | 80 | 49.18 | 76 | 173.55 | 88 | -7 | 8 | 12 |
| **orf17** | *iap-3* | e | reg | 174.63 | 26 | 75.17 | 29 | 232.17 | 23 | 879.67 | 33 | 7 | 4 | 10 |
| **orf18** | *odv-e56* | e, l | struc | 7.82 | 121 | 5.18 | 118 | 12.09 | 121 | 55.65 | 121 | 0 | 3 | 0 |
| **orf19** | *orf15R* | e |  | 79.11 | 48 | 37.91 | 52 | 94.15 | 51 | 482.6 | 49 | 1 | -3 | -2 |
| **orf20** | *orf16L* | l |  | 3.37 | 135 | 4.4 | 121 | 9.93 | 126 | 29.97 | 133 | -2 | 12 | 7 |
| **orf21** | *orf17L* |  |  | 48.03 | 61 | 28.81 | 63 | 53.82 | 69 | 138.09 | 97 | **36** | **34** | **28** |
| **orf22** | *orf17R* | l | struc | 3.86 | 133 | 3.64 | 123 | 8.01 | 131 | 43.38 | 129 | -4 | 6 | -2 |
| **orf23** | *pe/pp34* | e, l | struc | 15.26 | 106 | 24.11 | 66 | 31.04 | 99 | 141.69 | 96 | -10 | **30** | -3 |
| **orf24** | *pe38* | e | reg | 95.22 | 41 | 41.53 | 48 | 116.22 | 43 | 429.39 | 51 | 10 | 3 | 8 |
| **orf25** |  | e |  | 49.69 | 57 | 0 | 138 | 6.91 | 133 | 2.38 | 142 | **85** | 4 | 9 |
| **orf26** |  | l |  | 5.44 | 127 | 6.31 | 111 | 6.54 | 134 | 21.9 | 135 | 8 | **24** | 1 |
| **orf27** |  | e |  | 34.48 | 77 | 18.82 | 78 | 45.23 | 81 | 113.75 | 104 | **27** | **26** | **23** |
| **orf28/29** | *1* | e |  | 22.4 | 96 | 17.91 | 83 | 31.31 | 98 | 147.4 | 94 | -2 | 11 | -4 |
| **orf30** |  | e |  | 181.01 | 25 | 62.06 | 35 | 214.66 | 26 | 912.97 | 31 | 6 | -4 | 5 |
| **orf31** | *f-protein* | e | struc | 22.35 | 97 | 10.6 | 103 | 32.61 | 96 | 220.47 | 80 | -17 | **-23** | -16 |
| **orf32** |  | l |  | 246.77 | 18 | 136.14 | 14 | 344.22 | 15 | 1516.64 | 17 | -1 | 3 | 2 |
| **orf33** |  | l |  | 17.18 | 102 | 8.6 | 105 | 16.1 | 115 | 98.91 | 108 | 6 | 3 | -7 |
| **orf34** |  | e |  | 6.26 | 124 | 0 | 138 | 3.85 | 137 | 31.18 | 132 | 8 | -6 | -5 |
| **orf35** | *pif-3* | e, l | struc | 1.81 | 138 | 3 | 126 | 7.26 | 132 | 15.69 | 138 | 0 | 12 | 6 |
| **orf36b** |  | e |  | 0.81 | 140 | 0 | 138 | 3.11 | 138 | 13.42 | 139 | -1 | 1 | 1 |
| **orf36a** |  | e |  | 0 | 142 | 0 | 138 | 0 | 142 | 7.74 | 140 | -2 | 2 | -2 |
| **orf37** | *odv-e66* | l | struc | 0.8 | 141 | 0.31 | 137 | 1.53 | 140 | 7.38 | 141 | 0 | 4 | 1 |
| **orf39** |  | l |  | 21.56 | 99 | 6.52 | 110 | 19.12 | 112 | 107.2 | 106 | 7 | -4 | -6 |
| **orf40** |  |  |  | 140.03 | 30 | 52.39 | 39 | 158.3 | 36 | 812.44 | 36 | 6 | -3 | 0 |
| **orf41** | *lef-2* | e | reg | 49.69 | 56 | 46.91 | 41 | 83.2 | 58 | 357.75 | 59 | 3 | 18 | 1 |
| **orf42** | *orf35a* | e |  | 28.14 | 81 | 22.22 | 70 | 34.33 | 93 | 127.04 | 99 | 18 | **29** | 6 |
| **orf43** |  | e, l |  | 46.66 | 65 | 35.08 | 56 | 89.2 | 52 | 403.51 | 53 | -12 | -3 | 1 |
| **orf44** | *orf36L* | e, l |  | 811.49 | 3 | 456.04 | 3 | 1080.08 | 3 | 4651.25 | 3 | 0 | 0 | 0 |
| **orf45** |  | e |  | 437.89 | 7 | 235.72 | 6 | 538.83 | 7 | 2729.45 | 6 | -1 | 0 | -1 |
| **orf46** | *mp-nase* |  | struc | 39.13 | 72 | 22.16 | 71 | 49.29 | 75 | 399.87 | 54 | -18 | -17 | **-21** |
| **orf47** | *p13* | l | struc | 9.02 | 115 | 3.41 | 124 | 9.62 | 127 | 43.78 | 128 | 13 | 4 | 1 |
| **orf48** | *pif-1* |  | struc | 9.72 | 114 | 4.02 | 122 | 10.19 | 124 | 84.11 | 111 | -3 | -11 | -13 |
| **orf49** |  | l |  | 8.41 | 119 | 6.21 | 113 | 17.05 | 114 | 40.81 | 130 | 11 | 17 | 16 |
| **orf50/51** |  | l |  | 40.34 | 69 | 29.6 | 61 | 56.51 | 68 | 314.09 | 64 | -5 | 3 | -4 |
| **orf52b** | *ac106/107* | l |  | 3.7 | 134 | 0.95 | 133 | 6.28 | 135 | 20.3 | 136 | 2 | 3 | 1 |
| **orf52a** |  | e |  | 8.85 | 116 | 2.33 | 128 | 12.56 | 118 | 62.05 | 118 | 2 | -10 | 0 |
| **orf53** | *ac110* | e |  | 16.22 | 105 | 11.76 | 101 | 24.55 | 104 | 126.31 | 100 | -5 | -1 | -4 |
| **orf54** | *ubiquitin* |  | aux | 426.27 | 9 | 194.12 | 9 | 606.53 | 5 | 2480.54 | 9 | 0 | 0 | 4 |
| **orf55** | *odv-ec43* | e, l | struc | 28.83 | 79 | 15.58 | 89 | 41.92 | 85 | 237.81 | 73 | -6 | -16 | -12 |
| **orf56** | *ac108* | l | struc | 48.27 | 60 | 29.64 | 60 | 60.61 | 65 | 306.42 | 66 | 6 | 6 | 1 |
| **orf57** | *pp31/39K* | e | reg | 1242.81 | 2 | 754.88 | 2 | 1710.14 | 2 | 9698.41 | 2 | 0 | 0 | 0 |
| **orf58** | *lef-11* |  | reg | 248.07 | 16 | 78.54 | 28 | 181.98 | 30 | 1863.94 | 13 | -3 | -15 | **-17** |
| **orf59** | *sod* | l | aux | 8.59 | 117 | 0.87 | 134 | 10.95 | 123 | 107.6 | 105 | -12 | **-29** | **-18** |
| **orf60** | *p74* | l | struc | 10.67 | 113 | 5.52 | 116 | 20.59 | 109 | 71.86 | 116 | 3 | 0 | 7 |
| **orf61** |  | l |  | 27.29 | 85 | 29.22 | 62 | 50.83 | 73 | 196.49 | 86 | 1 | **24** | 13 |
| **orf62** |  | e, l |  | 67.99 | 52 | 132.24 | 18 | 101.31 | 48 | 233.02 | 76 | **24** | **58** | **28** |
| **orf63** | *bro* | e | reg | 47.03 | 63 | 18.52 | 79 | 26.01 | 101 | 122.8 | 102 | **39** | **23** | 1 |
| **orf64** |  | e, l |  | 182.62 | 24 | 132.72 | 16 | 325.92 | 17 | 824.08 | 35 | 11 | 19 | **18** |
| **orf65** |  | l |  | 6.83 | 123 | 5.76 | 114 | 22.16 | 107 | 50.76 | 125 | 2 | 11 | **18** |
| **orf66** | *ptp-2* |  | struc | 297.46 | 13 | 109.03 | 21 | 533.94 | 8 | 1570.55 | 16 | 3 | -5 | 8 |
| **orf67** |  | l |  | 83 | 45 | 19.65 | 77 | 65.48 | 63 | 430.93 | 50 | 5 | **-27** | -13 |
| **orf68** | *p47/pif-5* | e | reg | 28.78 | 80 | 15.75 | 87 | 49.17 | 77 | 228.31 | 77 | -3 | -10 | 0 |
| **orf69** |  | l |  | 92.18 | 42 | 68.32 | 33 | 195.12 | 28 | 862.99 | 34 | -8 | 1 | 6 |
| **orf70** |  | l |  | 5.1 | 128 | 0.62 | 136 | 1.37 | 141 | 32.39 | 131 | 3 | -5 | -10 |
| **orf71** | *p24capsid* | l | struc | 18.51 | 101 | 13.56 | 95 | 35.07 | 91 | 221.69 | 79 | **-22** | -16 | -12 |
| **orf72** |  |  |  | 571.63 | 5 | 132.29 | 17 | 445.44 | 11 | 3947.93 | 4 | -1 | -13 | -7 |
| **orf73** | *38.7K* | e |  | 325.84 | 11 | 156.96 | 12 | 386.28 | 12 | 2665.06 | 7 | -4 | -5 | -5 |
| **orf74** | *lef-1* | e | reg | 53.48 | 55 | 17.58 | 84 | 69.22 | 62 | 409.07 | 52 | -3 | **-32** | -10 |
| **orf75** |  |  |  | 8.39 | 120 | 8.13 | 107 | 19.85 | 111 | 92.41 | 110 | -10 | 3 | -1 |
| **orf76** | *fgf-1* | e | aux | 86.46 | 44 | 46.2 | 42 | 86.19 | 54 | 645.28 | 41 | -3 | -1 | -13 |
| **orf77** |  | e |  | 233.15 | 19 | 48.76 | 40 | 215.53 | 25 | 1499.46 | 18 | -1 | **-22** | -7 |
| **orf78** |  | e |  | 42.5 | 68 | 15.17 | 93 | 47.49 | 79 | 239.57 | 72 | 4 | -21 | -7 |
| **orf79** |  | l |  | 11.08 | 112 | 6.61 | 109 | 22.59 | 106 | 97.2 | 109 | -3 | 0 | 3 |
| **orf80** | *lef-6* | e | reg | 117.89 | 34 | 55.37 | 36 | 134.09 | 40 | 803.6 | 37 | 3 | 1 | -3 |
| **orf81** | *dbp* | e | reg | 429.6 | 8 | 226.15 | 7 | 533.54 | 9 | 2478.8 | 10 | 2 | 3 | 1 |
| **orf82b** | *82a* | e |  | 79.15 | 47 | 41.55 | 47 | 114.86 | 44 | 640.86 | 42 | -5 | -5 | -2 |
| **orf82a** | *82b* | e |  | 48.61 | 59 | 23.95 | 67 | 72.91 | 60 | 362.35 | 58 | -1 | -9 | -2 |
| **orf83** | *p45(p48)* | e | struc | 12.08 | 110 | 5.76 | 114 | 21.01 | 108 | 77.11 | 115 | 5 | 1 | 7 |
| **orf84** | *p12* | l | struc | 48.78 | 58 | 27.24 | 65 | 63.32 | 64 | 328.96 | 63 | 5 | -2 | -1 |
| **orf85** | *bv/odv-c42 (p40)* | e, l | struc | 205.27 | 20 | 106.48 | 22 | 270.73 | 21 | 1138.26 | 23 | 3 | 1 | 2 |
| **orf86** | *p6.9* | l | struc | 3166.11 | 1 | 1256.29 | 1 | 3391.38 | 1 | 15598.96 | 1 | 0 | 0 | 0 |
| **orf87** | *lef-5* |  | reg | 38.03 | 74 | 16.13 | 86 | 49.77 | 74 | 284.35 | 67 | -7 | -19 | -7 |
| **orf88** | *38k* |  | struc | 32.5 | 78 | 22.78 | 69 | 51.35 | 71 | 236.09 | 74 | -4 | 5 | 3 |
| **orf89** | *pif-4* | l | struc | 4.29 | 132 | 0.71 | 135 | 10.16 | 125 | 54.98 | 122 | -10 | -13 | -3 |
| **orf90** | *helicase* | l | reg | 73.74 | 51 | 32.68 | 58 | 87.04 | 53 | 500.93 | 48 | -3 | -10 | -5 |
| **orf91** | *odv-e25* | e, l | struc | 4.88 | 129 | 4.85 | 119 | 17.16 | 113 | 83.86 | 112 | -17 | -7 | -1 |
| **orf92** | *p18* | l | struc | 1.53 | 139 | 1.42 | 131 | 1.56 | 139 | 22.24 | 134 | -5 | 3 | -5 |
| **orf93** | *p33* | e, l | struc | 5.72 | 125 | 1.83 | 130 | 8.29 | 129 | 46.26 | 126 | 1 | -4 | -3 |
| **orf94** | *iap* | l | reg | 166.98 | 27 | 67.55 | 34 | 181.65 | 31 | 1095.3 | 24 | -3 | -10 | -7 |
| **orf95** | *lef-4* | l | reg | 26.45 | 88 | 6.23 | 112 | 34.36 | 92 | 198.73 | 84 | -4 | **-28** | -8 |
| **orf96** | *vp39* | l | struc | 268.77 | 14 | 82.61 | 26 | 265.23 | 22 | 1796.53 | 14 | 0 | -12 | -8 |
| **orf97** | *odv-ec27* | e, l | struc | 39.37 | 71 | 33.1 | 57 | 85.67 | 55 | 398.98 | 55 | -16 | -2 | 0 |
| **orf98** | *ptp* | e | struc | 186.79 | 23 | 171.46 | 10 | 333.01 | 16 | 1351.95 | 19 | -4 | 9 | 3 |
| **orf99** |  | e |  | 27.42 | 83 | 13.42 | 97 | 33.82 | 95 | 170.37 | 89 | 6 | -8 | -6 |
| **orf100** |  | l |  | 5.47 | 126 | 2.11 | 129 | 12.2 | 120 | 57.08 | 120 | -6 | -9 | 0 |
| **orf101** | *vp91* | e, l | struc | 2.54 | 137 | 1.21 | 132 | 4.66 | 136 | 16.77 | 137 | 0 | 5 | 1 |
| **orf102** | *tlp20* | l |  | 24.27 | 90 | 15.4 | 91 | 47.27 | 80 | 217.46 | 82 | -8 | -9 | 2 |
| **orf103** | *ac81* | l |  | 23.81 | 91 | 20.41 | 75 | 47.82 | 78 | 196.66 | 85 | -6 | 10 | 7 |
| **orf104** | *gp41* | l | struc | 8.57 | 118 | 3.18 | 125 | 12.88 | 116 | 65.95 | 117 | -1 | -8 | 1 |
| **orf105** | *ac78* | l |  | 13.14 | 108 | 2.65 | 127 | 20.38 | 110 | 81.68 | 113 | 5 | -14 | 3 |
| **orf106** |  | e, l |  | 74.34 | 50 | 36.19 | 54 | 101.08 | 49 | 532.69 | 47 | -3 | -7 | -2 |
| **orf107** |  |  |  | 4.68 | 130 | 5.42 | 117 | 8.94 | 128 | 60.1 | 119 | -11 | 2 | -9 |
| **orf108** | *ac75* | l | struc | 25.68 | 89 | 20.89 | 74 | 39.1 | 89 | 194.95 | 87 | -2 | 13 | -2 |
| **orf109** |  |  |  | 323.75 | 12 | 171.08 | 11 | 361.12 | 13 | 1223.94 | 22 | 10 | 11 | 9 |
| **orf110** |  | e |  | 106.08 | 35 | 90.56 | 24 | 151.77 | 37 | 888.34 | 32 | -3 | 8 | -5 |
| **orf111** | *dnapol* |  | reg | 23.76 | 92 | 12.6 | 99 | 31.6 | 97 | 219.21 | 81 | -11 | -18 | -16 |
| **orf112** | *desmoplakin* | e | struc | 34.9 | 76 | 17.95 | 81 | 39.1 | 88 | 243.31 | 71 | -5 | -10 | **-17** |
| **orf113** | *lef-3* | e | reg | 129.83 | 31 | 73.91 | 30 | 173.32 | 33 | 980.02 | 26 | -5 | -4 | -7 |
| **orf114** | *pif-6* | e | struc | 27.39 | 84 | 19.97 | 76 | 51.35 | 72 | 156 | 90 | 6 | 14 | **18** |
| **orf115** |  | e |  | 127.6 | 32 | 71.61 | 31 | 150.99 | 38 | 936.11 | 28 | -4 | -3 | -10 |
| **orf116** | *iap-5* | e | aux | 6.84 | 122 | 4.59 | 120 | 11.7 | 122 | 78.78 | 114 | -8 | -6 | -8 |
| **orf117** | *lef-9* | l | reg | 35.78 | 75 | 16.14 | 85 | 40.78 | 87 | 312.21 | 65 | -10 | -20 | **-22** |
| **orf118** | *fp25k* | l | struc | 125.45 | 33 | 43.4 | 44 | 174.32 | 32 | 927.64 | 29 | -4 | -15 | -3 |
| **orf119** |  | e |  | 203.63 | 21 | 105.36 | 23 | 279.69 | 19 | 1294.63 | 21 | 0 | -2 | 2 |
| **orf120** | *DNA ligase* |  | reg | 46.12 | 67 | 14.53 | 94 | 43.03 | 84 | 225.85 | 78 | 11 | -16 | -6 |
| **orf121** |  |  |  | 75.91 | 49 | 35.22 | 55 | 109.05 | 46 | 664.5 | 39 | -10 | -16 | -7 |
| **orf122** |  | e |  | 153.51 | 29 | 120.42 | 20 | 207.91 | 27 | 965.32 | 27 | -2 | 7 | 0 |
| **orf123** | *fgf* |  | aux | 261.82 | 15 | 133.36 | 15 | 276.17 | 20 | 1944.6 | 12 | -3 | -3 | -8 |
| **orf124** |  | e |  | 457.13 | 6 | 310.04 | 5 | 554.04 | 6 | 2086.02 | 11 | 5 | 6 | 5 |
| **orf125** | *alk-exo* | e | aux | 26.9 | 86 | 17.91 | 82 | 43.32 | 82 | 278.23 | 68 | -18 | -14 | -14 |
| **orf126** | *helicase-2* | e | reg | 104.47 | 36 | 71.22 | 32 | 165.35 | 35 | 758.7 | 38 | 2 | 6 | 3 |
| **orf127** | *rr1* | e | reg | 19.64 | 100 | 12.72 | 98 | 26.24 | 100 | 155.98 | 91 | -9 | -7 | -9 |
| **orf128** | *rr2a* |  | reg | 61.87 | 54 | 39.37 | 51 | 95.07 | 50 | 354.72 | 60 | 6 | 9 | 10 |
| **orf129/130** |  | e | reg | 166.39 | 28 | 82.12 | 27 | 229.27 | 24 | 1065.65 | 25 | -3 | -2 | 1 |
| **orf131** | *lef-8* | e | reg | 27.97 | 82 | 15.56 | 90 | 36.22 | 90 | 209.67 | 83 | 1 | -7 | -7 |
| **orf132** |  |  |  | 677.19 | 4 | 384.19 | 4 | 776.62 | 4 | 2626 | 8 | 4 | 4 | 4 |
| **orf133** |  | e |  | 80.45 | 46 | 42.08 | 45 | 118.6 | 42 | 581.66 | 45 | -1 | 0 | 3 |
| **orf134** |  | l |  | 46.35 | 66 | 36.99 | 53 | 51.51 | 70 | 233.14 | 75 | 9 | **22** | 5 |
| **orf135** |  | l |  | 247.78 | 17 | 145.83 | 13 | 358.59 | 14 | 1626.52 | 15 | -2 | 2 | 1 |
| **orf136** |  | l |  | 96.69 | 38 | 42.05 | 46 | 172.84 | 34 | 646.1 | 40 | 2 | -6 | 6 |
| **orf137** | *lef-10* | e, l | aux | 23.19 | 93 | 21.77 | 72 | 24.62 | 103 | 250.34 | 70 | **-23** | -2 | **-33** |
| **orf138** | *vp1054* | e | struc | 67.44 | 53 | 32.19 | 59 | 84.62 | 57 | 556.31 | 46 | -7 | -13 | -11 |
| **orf139** |  | e |  | 2.79 | 136 | 0 | 138 | 8.28 | 130 | 50.8 | 124 | -12 | -14 | -6 |
| **orf140** | *fgf-3* | e | aux | 425.49 | 10 | 198.72 | 8 | 447.6 | 10 | 2926.86 | 5 | -5 | -3 | -5 |
| **orf141** | *egt* | e | aux | 96.3 | 40 | 55.13 | 37 | 120.63 | 41 | 921.93 | 30 | -10 | -7 | -11 |
| **orf142** |  | e |  | 11.69 | 111 | 15.25 | 92 | 59.59 | 66 | 125.69 | 101 | -10 | 9 | **35** |
| **orf143** |  |  |  | 39.72 | 70 | 40.19 | 49 | 111.02 | 45 | 268.98 | 69 | -1 | 20 | **24** |
