# Supplementary material for: Transcriptome analysis of CpGV in midguts of type II resistant codling moth larvae and identification of contaminant infections by SNP mapping of RNA-Seq data
Source: J Virol. 2024 Jun 27;98(7):e00537-24. doi: 10.1128/jvi.00537-24 (PMC11265400; doi:10.1128/jvi.00537-24)
Supplement: Table S6 — Statistical analysis of TPM ratio. [file jvi.00537-24-s0008.docx]

**TABLE S6** Statistical analysis of the calculated TPM ratios for the sample groups M1-M3, S1 and S2S3. Given are the standard deviation of the mean (STDEV) and the maximum (Max) and minimum (Min) value of the ratios. The ratio values outside the 90% percentile (>90%) are given on the bottom line.

|  | **M1-M3** | **S1** | **S2S3** |
| --- | --- | --- | --- |
| **Mean** | 7.38 | 14.82 | 5.14 |
| **STDEV** | 2.62 | 12.73 | 2.39 |
| **Max** | 18.23 | 124.16 | 23.66 |
| **Min** | 0.05 | 1.76 | 0.34 |
| **>90%** | < 3.08, > 11.68 | < 6.12, > 35.76 | < 1.22, > 9.07 |
