# Supplementary material for: Transcriptome analysis of CpGV in midguts of type II resistant codling moth larvae and identification of contaminant infections by SNP mapping of RNA-Seq data
Source: J Virol. 2024 Jun 27;98(7):e00537-24. doi: 10.1128/jvi.00537-24 (PMC11265400; doi:10.1128/jvi.00537-24)
Supplement: Table S7 — Ratio of TPM value compared with CpGV-E2. [file jvi.00537-24-s0009.docx]

**TABLE S7:** TPM values and their ratios of different RNA-Seq samples of CpGV-M (M1-M3) and CpGV-S (S1, S2S3) compared with CpGV-E2 (E1-E3). Given are the open reading frame number (ID) and the gene name, promoter motif(s) and putative function (as far as known) (see Wennmann et al., 2021. Journal of General Virology 102(3), 001566. DOI 10.1099/jgv.0.001566) and the calculated ratios (= TPM(E1-E3)orf[i]/TPM[X]orf [i], with i = 1 to 143 and X = M1-M3, S1, S2S3). The ratios given in orange bold font were significantly upregulated whereas blue bold font indicates downregulation compared to CpGV-E2**.** The TPM <1 means no expression. Please note that significant ratio changes have different ranges for M1-M3, S1 and S2S3 (see Table S6).

| **Ratios of viral gene expression in all groups** | | | | | | | | | | |
| --- | --- | --- | --- | --- | --- | --- | --- | --- | --- | --- |
|  |  |  |  | **Every gene TPM value in all groups** | | | | **Ratio of TPM value compared with CpGV-E2** | | |
| **ID** | **Gene** | **Promoter** | **Function** | **M1-M3** | **S1** | **S2S3** | **E1-E3** | **M1-M3** | **S1** | **S2S3** |
| **orf1** | *granulin* | l | struc | 16.36 | 15.74 | 41.45 | 116.26 | 7.11 | 7.39 | 2.8 |
| **orf2** |  | e, l |  | 16.75 | 9.22 | 25.69 | 104.6 | 6.24 | 11.34 | 4.07 |
| **orf3** | *pk1* | e | struc | 96.36 | 85.21 | 188.37 | 582.56 | 6.05 | 6.84 | 3.09 |
| **orf4** |  | e, l |  | 99.38 | 45.13 | 108.55 | 626.53 | 6.3 | 13.88 | 5.77 |
| **orf5** |  |  |  | 38.65 | 39.84 | 71.14 | 369.52 | 9.56 | 9.27 | 5.19 |
| **orf6** |  | l |  | 47.16 | 27.35 | 57.95 | 348.57 | 7.39 | 12.75 | 6.01 |
| **orf7** | *ie-1* | e | reg | 14.84 | 12.49 | 23.05 | 130.05 | 8.77 | 10.41 | 5.64 |
| **orf8** | *ac146* | e | struc | 196.43 | 120.63 | 301.83 | 1297.85 | 6.61 | 10.76 | 4.3 |
| **orf9** | *ac145* | e, l | struc | 4.38 | 7.91 | 12.42 | 44.31 | 10.11 | **5.6** | 3.57 |
| **orf10** | *chitinase* | e | aux | 46.85 | 23.24 | 85.56 | 365.18 | 7.79 | 15.71 | 4.27 |
| **orf11** | *cathepsin* | l | aux | 26.63 | 21.74 | 72.99 | 154.33 | 5.8 | 7.1 | 2.11 |
| **orf12** |  | l |  | 86.64 | 54.67 | 142.06 | 332.51 | 3.84 | **6.08** | 2.34 |
| **orf13** | *gp37* | l | aux | 22.87 | 10.98 | 33.92 | 155.03 | 6.78 | 14.12 | 4.57 |
| **orf14** | *odv-e18* | l | struc | 22.21 | 13.56 | 43.21 | 146.02 | 6.57 | 10.77 | 3.38 |
| **orf15** | *p49* | e, l | struc | 12.15 | 8.3 | 12.72 | 52.98 | 4.36 | 6.38 | 4.17 |
| **orf16** |  | e |  | 22.45 | 18.14 | 49.18 | 173.55 | 7.73 | 9.57 | 3.53 |
| **orf17** | *iap-3* | e | reg | 174.63 | 75.17 | 232.17 | 879.67 | 5.04 | 11.7 | 3.79 |
| **orf18** | *odv-e56* | e, l | struc | 7.82 | 5.18 | 12.09 | 55.65 | 7.12 | 10.74 | 4.6 |
| **orf19** | *orf15R* | e |  | 79.11 | 37.91 | 94.15 | 482.6 | 6.1 | 12.73 | 5.13 |
| **orf20** | *orf16L* | l |  | 3.37 | 4.4 | 9.93 | 29.97 | 8.9 | 6.82 | 3.02 |
| **orf21** | *orf17L* |  |  | 48.03 | 28.81 | 53.82 | 138.09 | **2.87** | **4.79** | 2.57 |
| **orf22** | *orf17R* | l | struc | 3.86 | 3.64 | 8.01 | 43.38 | 11.25 | 11.91 | 5.42 |
| **orf23** | *pe/pp34* | e, l | struc | 15.26 | 24.11 | 31.04 | 141.69 | 9.28 | **5.88** | 4.56 |
| **orf24** | *pe38* | e | reg | 95.22 | 41.53 | 116.22 | 429.39 | 4.51 | 10.34 | 3.69 |
| **orf25** |  | e |  | 49.69 | 0 | 6.91 | 2.38 | **0.05** | / | **0.34** |
| **orf26** |  | l |  | 5.44 | 6.31 | 6.54 | 21.9 | 4.03 | **3.47** | 3.35 |
| **orf27** |  | e |  | 34.48 | 18.82 | 45.23 | 113.75 | 3.3 | **6.04** | 2.51 |
| **orf28/29** | *1* | e |  | 22.4 | 17.91 | 31.31 | 147.4 | 6.58 | 8.23 | 4.71 |
| **orf30** |  | e |  | 181.01 | 62.06 | 214.66 | 912.97 | 5.04 | 14.71 | 4.25 |
| **orf31** | *f-protein* | e | struc | 22.35 | 10.6 | 32.61 | 220.47 | 9.86 | 20.8 | 6.76 |
| **orf32** |  | l |  | 246.77 | 136.14 | 344.22 | 1516.64 | 6.15 | 11.14 | 4.41 |
| **orf33** |  | l |  | 17.18 | 8.6 | 16.1 | 98.91 | 5.76 | 11.51 | 6.14 |
| **orf34** |  | e |  | 6.26 | 0 | 3.85 | 31.18 | 4.98 | / | 8.09 |
| **orf35** | *pif-3* | e, l | struc | 1.81 | 3 | 7.26 | 15.69 | 8.66 | **5.23** | 2.16 |
| **orf36b** |  | e |  | 0.81 | 0 | 3.11 | 13.42 | **16.48** | / | 4.31 |
| **orf36a** |  | e |  | 0 | 0 | 0 | 7.74 | / | / | / |
| **orf37** | *odv-e66* | l | struc | 0.8 | 0.31 | 1.53 | 7.38 | 9.23 | 23.87 | 4.83 |
| **orf39** |  | l |  | 21.56 | 6.52 | 19.12 | 107.2 | 4.97 | 16.43 | 5.61 |
| **orf40** |  |  |  | 140.03 | 52.39 | 158.3 | 812.44 | 5.8 | 15.51 | 5.13 |
| **orf41** | *lef-2* | e | reg | 49.69 | 46.91 | 83.2 | 357.75 | 7.2 | 7.63 | 4.3 |
| **orf42** | *orf35a* | e |  | 28.14 | 22.22 | 34.33 | 127.04 | 4.52 | **5.72** | 3.7 |
| **orf43** |  | e, l |  | 46.66 | 35.08 | 89.2 | 403.51 | 8.65 | 11.5 | 4.52 |
| **orf44** | *orf36L* | e, l |  | 811.49 | 456.04 | 1080.08 | 4651.25 | 5.73 | 10.2 | 4.31 |
| **orf45** |  | e |  | 437.89 | 235.72 | 538.83 | 2729.45 | 6.23 | 11.58 | 5.07 |
| **orf46** | *mp-nase* |  | struc | 39.13 | 22.16 | 49.29 | 399.87 | 10.22 | 18.04 | 8.11 |
| **orf47** | *p13* | l | struc | 9.02 | 3.41 | 9.62 | 43.78 | 4.86 | 12.82 | 4.55 |
| **orf48** | *pif-1* |  | struc | 9.72 | 4.02 | 10.19 | 84.11 | 8.65 | 20.94 | 8.26 |
| **orf49** |  | l |  | 8.41 | 6.21 | 17.05 | 40.81 | 4.85 | 6.58 | 2.39 |
| **orf50/51** |  | l |  | 40.34 | 29.6 | 56.51 | 314.09 | 7.79 | 10.61 | 5.56 |
| **orf52b** | *ac106/107* | l |  | 3.7 | 0.95 | 6.28 | 20.3 | 5.49 | 21.31 | 3.23 |
| **orf52a** |  | e |  | 8.85 | 2.33 | 12.56 | 62.05 | 7.01 | 26.59 | 4.94 |
| **orf53** | *ac110* | e |  | 16.22 | 11.76 | 24.55 | 126.31 | 7.79 | 10.74 | 5.14 |
| **orf54** | *ubiquitin* |  | aux | 426.27 | 194.12 | 606.53 | 2480.54 | 5.82 | 12.78 | 4.09 |
| **orf55** | *odv-ec43* | e, l | struc | 28.83 | 15.58 | 41.92 | 237.81 | 8.25 | 15.26 | 5.67 |
| **orf56** | *ac108* | l | struc | 48.27 | 29.64 | 60.61 | 306.42 | 6.35 | 10.34 | 5.06 |
| **orf57** | *pp31/39K* | e | reg | 1242.81 | 754.88 | 1710.14 | 9698.41 | 7.8 | 12.85 | 5.67 |
| **orf58** | *lef-11* |  | reg | 248.07 | 78.54 | 181.98 | 1863.94 | 7.51 | 23.73 | **10.24** |
| **orf59** | *sod* | l | aux | 8.59 | 0.87 | 10.95 | 107.6 | **12.52** | **124.16** | **9.83** |
| **orf60** | *p74* | l | struc | 10.67 | 5.52 | 20.59 | 71.86 | 6.73 | 13.02 | 3.49 |
| **orf61** |  | l |  | 27.29 | 29.22 | 50.83 | 196.49 | 7.2 | 6.72 | 3.87 |
| **orf62** |  | e, l |  | 67.99 | 132.24 | 101.31 | 233.02 | 3.43 | **1.76** | 2.3 |
| **orf63** | *bro* | e | reg | 47.03 | 18.52 | 26.01 | 122.8 | **2.61** | 6.63 | 4.72 |
| **orf64** |  | e, l |  | 182.62 | 132.72 | 325.92 | 824.08 | 4.51 | 6.21 | 2.53 |
| **orf65** |  | l |  | 6.83 | 5.76 | 22.16 | 50.76 | 7.43 | 8.81 | 2.29 |
| **orf66** | *ptp-2* |  | struc | 297.46 | 109.03 | 533.94 | 1570.55 | 5.28 | 14.41 | 2.94 |
| **orf67** |  | l |  | 83 | 19.65 | 65.48 | 430.93 | 5.19 | 21.93 | 6.58 |
| **orf68** | *p47/pif-5* | e | reg | 28.78 | 15.75 | 49.17 | 228.31 | 7.93 | 14.5 | 4.64 |
| **orf69** |  | l |  | 92.18 | 68.32 | 195.12 | 862.99 | 9.36 | 12.63 | 4.42 |
| **orf70** |  | l |  | 5.1 | 0.62 | 1.37 | 32.39 | 6.35 | **52** | 23.66 |
| **orf71** | *p24capsid* | l | struc | 18.51 | 13.56 | 35.07 | 221.69 | **11.98** | 16.35 | 6.32 |
| **orf72** |  |  |  | 571.63 | 132.29 | 445.44 | 3947.93 | 6.91 | 29.84 | 8.86 |
| **orf73** | *38.7K* | e |  | 325.84 | 156.96 | 386.28 | 2665.06 | 8.18 | 16.98 | 6.9 |
| **orf74** | *lef-1* | e | reg | 53.48 | 17.58 | 69.22 | 409.07 | 7.65 | 23.27 | 5.91 |
| **orf75** |  |  |  | 8.39 | 8.13 | 19.85 | 92.41 | 11.02 | 11.37 | 4.65 |
| **orf76** | *fgf-1* | e | aux | 86.46 | 46.2 | 86.19 | 645.28 | 7.46 | 13.97 | 7.49 |
| **orf77** |  | e |  | 233.15 | 48.76 | 215.53 | 1499.46 | 6.43 | 30.75 | 6.96 |
| **orf78** |  | e |  | 42.5 | 15.17 | 47.49 | 239.57 | 5.64 | 15.8 | 5.04 |
| **orf79** |  | l |  | 11.08 | 6.61 | 22.59 | 97.2 | 8.77 | 14.71 | 4.3 |
| **orf80** | *lef-6* | e | reg | 117.89 | 55.37 | 134.09 | 803.6 | 6.82 | 14.51 | 5.99 |
| **orf81** | *dbp* | e | reg | 429.6 | 226.15 | 533.54 | 2478.8 | 5.77 | 10.96 | 4.65 |
| **orf82b** | *82a* | e |  | 79.15 | 41.55 | 114.86 | 640.86 | 8.1 | 15.43 | 5.58 |
| **orf82a** | *82b* | e |  | 48.61 | 23.95 | 72.91 | 362.35 | 7.45 | 15.13 | 4.97 |
| **orf83** | *p45 (p48)* | e | struc | 12.08 | 5.76 | 21.01 | 77.11 | 6.38 | 13.38 | 3.67 |
| **orf84** | *p12* | l | struc | 48.78 | 27.24 | 63.32 | 328.96 | 6.74 | 12.08 | 5.2 |
| **orf85** | *bv/odv-c42 (p40)* | e, l | struc | 205.27 | 106.48 | 270.73 | 1138.26 | 5.55 | 10.69 | 4.2 |
| **orf86** | *p6.9* | l | struc | 3166.11 | 1256.29 | 3391.38 | 15598.96 | 4.93 | 12.42 | 4.6 |
| **orf87** | *lef-5* |  | reg | 38.03 | 16.13 | 49.77 | 284.35 | 7.48 | 17.63 | 5.71 |
| **orf88** | *38k* |  | struc | 32.5 | 22.78 | 51.35 | 236.09 | 7.26 | 10.36 | 4.6 |
| **orf89** | *pif-4* | l | struc | 4.29 | 0.71 | 10.16 | 54.98 | **12.8** | **77.28** | 5.41 |
| **orf90** | *helicase* | l | reg | 73.74 | 32.68 | 87.04 | 500.93 | 6.79 | 15.33 | 5.76 |
| **orf91** | *odv-e25* | e, l | struc | 4.88 | 4.85 | 17.16 | 83.86 | **17.2** | 17.3 | 4.89 |
| **orf92** | *p18* | l | struc | 1.53 | 1.42 | 1.56 | 22.24 | **14.5** | 15.63 | **14.22** |
| **orf93** | *p33* | e, l | struc | 5.72 | 1.83 | 8.29 | 46.26 | 8.09 | 25.29 | 5.58 |
| **orf94** | *iap* | l | reg | 166.98 | 67.55 | 181.65 | 1095.3 | 6.56 | 16.22 | 6.03 |
| **orf95** | *lef-4* | l | reg | 26.45 | 6.23 | 34.36 | 198.73 | 7.51 | 31.9 | 5.78 |
| **orf96** | *vp39* | l | struc | 268.77 | 82.61 | 265.23 | 1796.53 | 6.68 | 21.75 | 6.77 |
| **orf97** | *odv-ec27* | e, l | struc | 39.37 | 33.1 | 85.67 | 398.98 | 10.13 | 12.05 | 4.66 |
| **orf98** | *ptp* | e | struc | 186.79 | 171.46 | 333.01 | 1351.95 | 7.24 | 7.88 | 4.06 |
| **orf99** |  | e |  | 27.42 | 13.42 | 33.82 | 170.37 | 6.21 | 12.69 | 5.04 |
| **orf100** |  | l |  | 5.47 | 2.11 | 12.2 | 57.08 | 10.44 | 26.99 | 4.68 |
| **orf101** | *vp91* | e, l | struc | 2.54 | 1.21 | 4.66 | 16.77 | 6.61 | 13.84 | 3.6 |
| **orf102** | *tlp20* | l |  | 24.27 | 15.4 | 47.27 | 217.46 | 8.96 | 14.12 | 4.6 |
| **orf103** | *ac81* | l |  | 23.81 | 20.41 | 47.82 | 196.66 | 8.26 | 9.64 | 4.11 |
| **orf104** | *gp41* | l | struc | 8.57 | 3.18 | 12.88 | 65.95 | 7.7 | 20.74 | 5.12 |
| **orf105** | *ac78* | l |  | 13.14 | 2.65 | 20.38 | 81.68 | 6.22 | 30.83 | 4.01 |
| **orf106** |  | e, l |  | 74.34 | 36.19 | 101.08 | 532.69 | 7.17 | 14.72 | 5.27 |
| **orf107** |  |  |  | 4.68 | 5.42 | 8.94 | 60.1 | **12.85** | 11.08 | 6.72 |
| **orf108** | *ac75* | l | struc | 25.68 | 20.89 | 39.1 | 194.95 | 7.59 | 9.33 | 4.99 |
| **orf109** |  |  |  | 323.75 | 171.08 | 361.12 | 1223.94 | 3.78 | 7.15 | 3.39 |
| **orf110** |  | e |  | 106.08 | 90.56 | 151.77 | 888.34 | 8.37 | 9.81 | 5.85 |
| **orf111** | *dnapol* |  | reg | 23.76 | 12.6 | 31.6 | 219.21 | 9.23 | 17.4 | 6.94 |
| **orf112** | *desmoplakin* | e | struc | 34.9 | 17.95 | 39.1 | 243.31 | 6.97 | 13.55 | 6.22 |
| **orf113** | *lef-3* | e | reg | 129.83 | 73.91 | 173.32 | 980.02 | 7.55 | 13.26 | 5.65 |
| **orf114** | *pif-6* | e | struc | 27.39 | 19.97 | 51.35 | 156 | 5.7 | 7.81 | 3.04 |
| **orf115** |  | e |  | 127.6 | 71.61 | 150.99 | 936.11 | 7.34 | 13.07 | 6.2 |
| **orf116** | *iap-5* | e | aux | 6.84 | 4.59 | 11.7 | 78.78 | 11.52 | 17.15 | 6.73 |
| **orf117** | *lef-9* | l | reg | 35.78 | 16.14 | 40.78 | 312.21 | 8.73 | 19.35 | 7.66 |
| **orf118** | *fp25k* | l | struc | 125.45 | 43.4 | 174.32 | 927.64 | 7.39 | 21.37 | 5.32 |
| **orf119** |  | e |  | 203.63 | 105.36 | 279.69 | 1294.63 | 6.36 | 12.29 | 4.63 |
| **orf120** | *DNA ligase* |  | reg | 46.12 | 14.53 | 43.03 | 225.85 | 4.9 | 15.54 | 5.25 |
| **orf121** |  |  |  | 75.91 | 35.22 | 109.05 | 664.5 | 8.75 | 18.87 | 6.09 |
| **orf122** |  | e |  | 153.51 | 120.42 | 207.91 | 965.32 | 6.29 | 8.02 | 4.64 |
| **orf123** | *fgf* |  | aux | 261.82 | 133.36 | 276.17 | 1944.6 | 7.43 | 14.58 | 7.04 |
| **orf124** |  | e |  | 457.13 | 310.04 | 554.04 | 2086.02 | 4.56 | 6.73 | 3.77 |
| **orf125** | *alk-exo* | e | aux | 26.9 | 17.91 | 43.32 | 278.23 | 10.34 | 15.54 | 6.42 |
| **orf126** | *helicase-2* | e | reg | 104.47 | 71.22 | 165.35 | 758.7 | 7.26 | 10.65 | 4.59 |
| **orf127** | *rr1* | e | reg | 19.64 | 12.72 | 26.24 | 155.98 | 7.94 | 12.26 | 5.94 |
| **orf128** | *rr2a* |  | reg | 61.87 | 39.37 | 95.07 | 354.72 | 5.73 | 9.01 | 3.73 |
| **orf129/130** |  | e | reg | 166.39 | 82.12 | 229.27 | 1065.65 | 6.4 | 12.98 | 4.65 |
| **orf131** | *lef-8* | e | reg | 27.97 | 15.56 | 36.22 | 209.67 | 7.5 | 13.47 | 5.79 |
| **orf132** |  |  |  | 677.19 | 384.19 | 776.62 | 2626 | 3.88 | 6.84 | 3.38 |
| **orf133** |  | e |  | 80.45 | 42.08 | 118.6 | 581.66 | 7.23 | 13.82 | 4.9 |
| **orf134** |  | l |  | 46.35 | 36.99 | 51.51 | 233.14 | 5.03 | 6.3 | 4.53 |
| **orf135** |  | l |  | 247.78 | 145.83 | 358.59 | 1626.52 | 6.56 | 11.15 | 4.54 |
| **orf136** |  | l |  | 96.69 | 42.05 | 172.84 | 646.1 | 6.68 | 15.36 | 3.74 |
| **orf137** | *lef-10* | e, l | aux | 23.19 | 21.77 | 24.62 | 250.34 | 10.8 | 11.5 | **10.17** |
| **orf138** | *vp1054* | e | struc | 67.44 | 32.19 | 84.62 | 556.31 | 8.25 | 17.28 | 6.57 |
| **orf139** |  | e |  | 2.79 | 0 | 8.28 | 50.8 | **18.23** | / | 6.13 |
| **orf140** | *fgf-3* | e | aux | 425.49 | 198.72 | 447.6 | 2926.86 | 6.88 | 14.73 | 6.54 |
| **orf141** | *egt* | e | aux | 96.3 | 55.13 | 120.63 | 921.93 | 9.57 | 16.72 | 7.64 |
| **orf142** |  | e |  | 11.69 | 15.25 | 59.59 | 125.69 | 10.75 | 8.24 | 2.11 |
| **orf143** |  |  |  | 39.72 | 40.19 | 111.02 | 268.98 | 6.77 | 6.69 | 2.42 |
